# LysSYL: a broad-spectrum phage endolysin targeting *Staphylococcus* species and eradicating *S. aureus* biofilms

**DOI:** 10.1186/s12934-024-02359-4

**Published:** 2024-03-25

**Authors:** He Liu, Xuemei Wei, Zhefen Wang, Xiaonan Huang, Mengyang Li, Zhen Hu, Kexin Zhang, Qiwen Hu, Huagang Peng, Weilong Shang, Yi Yang, Yuting Wang, Shuguang Lu, Xiancai Rao

**Affiliations:** 1 Department of Microbiology, College of Basic Medical Sciences, Key Laboratory of Microbial Engineering Under the Educational Committee in Chongqing, Army Medical University, Chongqing, 400038 China; 2 Medical Research Institute, Southwest University, Chongqing, 400700 China; 3 Department of Neurology, The First Affiliated Hospital of Kunming Medical University, Kunming, 650032 Yunnan Province China; 4 Department of Microbiology, School of Medicine, Chongqing University, Chongqing, 400044 China; 5 Immunology Research Center, Medical Research Institute, Southwest University, Chongqing, 400700 China

**Keywords:** Phage, Endolysin, *Staphylococcus aureus*, Biofilms, Persisters

## Abstract

**Background:**

*Staphylococcus aureus* and its single or mixed biofilm infections seriously threaten global public health. Phage therapy, which uses active phage particles or phage-derived endolysins, has emerged as a promising alternative strategy to antibiotic treatment. However, high-efficient phage therapeutic regimens have yet to be established.

**Results:**

In this study, we used an enrichment procedure to isolate phages against methicillin-resistant *S. aureus* (MRSA) XN108. We characterized phage SYL, a new member of the *Kayvirus* genus, *Herelleviridae* family. The phage endolysin LysSYL was expressed. LysSYL demonstrated stability under various conditions and exhibited a broader range of efficacy against staphylococcal strains than its parent phage (100% vs. 41.7%). Moreover, dynamic live/dead bacterial observation demonstrated that LysSYL could completely lyse MRSA USA300 within 10 min. Scan and transmission electron microscopy revealed evident bacterial cell perforation and deformation. In addition, LysSYL displayed strong eradication activity against single- and mixed-species biofilms associated with *S. aureus*. It also had the ability to kill bacterial persisters, and proved highly effective in eliminating persistent *S. aureus* when combined with vancomycin. Furthermore, LysSYL protected BALB/c mice from lethal *S. aureus* infections. A single-dose treatment with 50 mg/kg of LysSYL resulted in a dramatic reduction in bacterial loads in the blood, liver, spleen, lungs, and kidneys of a peritonitis mouse model, which resulted in rescuing 100% of mice challenged with 10^8^ colony forming units of *S. aureus* USA300.

**Conclusions:**

Overall, the data provided in this study highlight the strong therapeutic potential of endolysin LysSYL in combating staphylococcal infections, including mono- and mixed-species biofilms related to *S. aureus*.

**Supplementary Information:**

The online version contains supplementary material available at 10.1186/s12934-024-02359-4.

## Background

Antimicrobial resistance (AMR) in clinically important pathogens threatens the effective prevention and treatment of the increasingly prevalent infections [1]. A large-scale survey revealed that 33 bacterial pathogens were responsible for approximately 13.6% of all global deaths in 2019 [2]. The top five pathogens (*Staphylococcus aureus*, *Escherichia coli*, *Streptococcus pneumoniae*, *Klebsiella pneumoniae*, and *Pseudomonas aeruginosa*) accounted for 54.9% of the deaths attributed to bacterial infections [2]. *S. aureus*, as a prominent human pathogen, can cause various infections, which range from skin/soft tissue infections to life-threatening pneumonia, bacteremia, and sepsis [3]. One of the major reasons for *S. aureus* becoming a leading cause of death is its capability to rapidly acquire resistance. Methicillin-resistant *S. aureus* (MRSA) emerged as a multidrug-resistant (MDR) bacterium since its discovery in 1961, only 2 years after the introduction of β-lactamase-resistant methicillin into clinical use [4]. Vancomycin (VAN), which is a glycopeptide antibiotic, proved effective against MRSA infections and has been widely used in clinical settings since the early 1980s [5]. However, *S. aureus* strains with reduced VAN susceptibility, such as VAN-intermediate *S. aureus* (VISA) isolate Mu50 (MIC = 8 µg/mL) and the heterogeneous VISA (hVISA) isolate Mu3 (MIC = 3 µg/mL), were first discovered in Japan in 1997 and subsequently reported globally [6, 7]. Moreover, *S. aureus* can express MDR phenotypes by developing biofilms, forming persisters, or exhibiting small-colony variants [8, 9].

Bacterial biofilms are monospecies or multispecies microbial communities in which microbes adhere to each other and to biotic or abiotic surface [8]. The US National Institutes of Health reported that over 80% of microbial infections in humans are associated with biofilms, which resulted in an estimated annual economic impact of $3,967 bn globally [10]. *S. aureus* forms mono-species biofilms to evade host immune responses, and biofilm-encased staphylococcal cells can be 10–1000 times more resistant to conventional antibiotics than their planktonic counterparts [8, 11], leading to recalcitrant and often chronic infections in clinic [12]. Moreover, *S. aureus* is notorious for its ability to generate mixed-species biofilms with other pathogens. Statistics of 80.9% of microbial biofilm cases in venous catheter infections are associated with *Acinetobacter baumannii*, *Staphylococcus* spp., *E. coli*, and *K. pneumoniae* [12]. In addition, the co-infections caused by *S. aureus* and *A. baumannii* predominate in 760 collected clinical samples during 2017 to 2019 [13]. The polymicrobial infections not only are more virulent than single-species infections but also lead to a chronic stage of infection [14]. Therefore, the development of novel antimicrobial alternatives to control drug-resistant *S. aureus* infections is urgently needed.

Bacteriophage (phage) therapy, which utilizes phage itself or its lytic elements, such as endolysins, has emerged as an important research area in the quest for alternatives to combat the antibiotic-resistance crisis [3, 15]. Phages in nature exhibit a high degree of diversity and abundance [16]. However, the limitations of strict host-specificity, complicated clinical evaluation, and an inadequate regulatory framework of phages hinder the widespread application of active phage therapy [17]. Endolysins were first described in the lysates of *S. aureus* in the early 1960s [18]. In general, endolysins from phages targeting Gram-positive bacteria feature an architecture comprising two different types of domains: an N-terminal catalytic domain (CD) and a C-terminal cell wall binding domain (CBD) [19]. However, the domains are variable in certain endolysins. Streptococcal endolysins λSA2 and PlySK1249 carry a central CBD separated by the two CDs; while endolysin Ply187 derived from *S. aureus* phage 187 contains two CDs but lacks a CBD [20, 21]. Moreover, the CD in endolysins may have distinct hydrolase activities, such as cysteine- and histidiane-dependent aminopeptidase/hydrolase (CHAP), N-acetylmuramoyl-L-alanine amidase (amidase 2), muramidase, or glucosaminidase [19]. Some endolysins carrying certain CDs are highly efficient hydrolases that degrade the peptidoglycan of bacterial cell walls to result in bacterial lysis and death [15]. Compared with active phages, endolysins offer rapid bactericidal activity and a relatively broad host spectrum [17, 22]. Treatment with natural or artificially constructed endolysins has shown promising results against Gram-positive bacterial infections [19, 23]. However, the establishment of highly effective phage therapeutic regimens is challenging.

In this study, an enrichment procedure was used to isolate phages targeting the MRSA strain XN108, and an *S. aureus* phage named SYL was characterized and genome-sequenced. The endolysin LysSYL was expressed in *E. coli*, and its lytic spectrum was determined. LysSYL demonstrated stability under diverse conditions and exhibited broad-spectrum activity in killing planktonic *S. aureus* cells, persisters, and biofilms. These findings highlight its potential as a promising antimicrobial agent for effectively addressing drug-resistant *S. aureus* infections.

## Materials and methods

### Bacterial strains and growth conditions

All strains used in this study are listed in Additional file 3: Table S1. *S. aureus* strain XN108 (MRSA and VISA) was isolated from a burn patient with skin wound infection and bacteremia [24]. Methicillin-sensitive *S. aureus* (MSSA) ATCC 25923, *A. baumannii* ATCC 19606, and *P. aeruginosa* PAO1 were purchased from China Center for Type Culture Collection. *E. coli* strains DH5a and BL21 (DE3) were purchased from TransGen Biotech (China). *S. aureus* strains were cultivated in brain heart infusion (BHI) medium (Oxoid) or BHI agar (BHIA). *E. coli, A. baumannii*, and *P. aeruginosa* strains were grown in Luria Bertani (LB) medium (Oxoid). When appropriate, the broth media or agar plates were supplemented with ampicillin (AMP, 100 µg/mL).

### Phage isolation with MRSA XN108 as the host

An enrichment procedure was performed to isolate *S. aureus* phages [25]. Briefly, a total of 20 sewage water samples (300 mL for each) collected from diverse locations were mixed and centrifuged at 10,000 ×g for 10 min to remove cells and cellular debris. Then, the supernatant was filtered through a 0.22 μm vacuum filter (Millipore, USA) to remove possible aggregates. Tangential flow was performed to concentrate the filtrate at least 10 times with a 100 kDa cut-off hollow fiber cartridge column (GE healthcare, USA). Subsequently, 200 mL *S. aureus* host XN108 culture with an optical density (OD) at 600 nm of 0.5 was added to the filtrate and cultured at 37 °C for 12 h. After that, the culture was filtered again through a 0.22 μm filter, and the supernatant was serially diluted and spotted on agar plates inoculated with XN108 host bacteria. After incubation at 37 °C overnight, single plaques with diverse lytic phenotypes were picked, and the subsequent plaque isolation was performed with double-agar layer method [26]. A phage that formed clear and big plaques on *S. aureus* XN108 lawn was designated as SYL.

### Observation of phage SYL with transmission electron microscope

Phage SYL was added to a culture of XN108 in the log phase growth, and incubated at 37 °C for 6 h. Then, the culture was centrifuged at 10,000 ×g for 5 min and filtered through a 0.22 μm filter. The resulting phage lysate was concentrated and purified by PEG8000 precipitation according to the method described previously [26]. The purified SYL phage particles were further purified by using CsCl gradient ultracentrifugation as described [26]. For transmission electron microscopy (TEM), the purified phage SYL was prefixed with 3% (v/v) glutaraldehyde, postfixed in 1% (m/v) osmium tetroxide (OsO_4_) for 2 h, dehydrated in series acetone, infiltrated in the ethoxyline 812, and embedded. The thin sections were prepared and stained with uranyl acetate and lead citrate, observed under a JEM-1400-FLASH TEM (JEOL, Japan).

### Phage genome sequencing and bioinformation analysis

The genomic DNA was extracted and purified from phage lysates using the method described previously [27]. The genome DNA was verified by 1% (m/v) agarose gel electrophoresis and sent to Beijing Tsingke Biotech Co., Ltd (China) for sequencing. The whole genome sequencing of phage SYL was performed using the Illumina NovaSeq platform with 150 bp average read length and a total of 23,867,094 reads were obtained. Then, the original sequence was assembled with the SPAdes v. 3.5.0 software package [24]. The results were analyzed using GeneMarkS (http://topaz.gatech.edu/ GeneMark) [28] and RAST (http://rast.nmpdr.org/) [27] to predict and annotate the protein-coding genes of the phage genome. The phage whole-.

genome map was constructed by Proksee (https://proksee.ca/) [29]. The phage SYL genome sequence has been annotated and submitted to the GenBank database (accession number: OP235318.1).

The entire genome sequences of SYL and its related phages (max BlastN score > 35,000) were downloaded from National Center for Biotechnology Information (NCBI). The phylogenetic analysis of the entire phage genome sequences was conducted using VICTOR (Virus Classification and Tree Building Online Resource, https://ggdc.dsmz.de/victor.php) with default parameters [30].

### Analysis of endolysin LysSYL

A protein (protein ID: UVD37129.1) encoded in phage SYL showed high similarity to that of a putative endolysin and named as LysSYL. The molecular weight and isoelectric point (pI) of the LysSYL were predicted by ProtParam (https://web.expasy.org/protparam/). The functional domain analysis of LysSYL was performed using the NCBI Conserved Domain Database (https://www.ncbi.nlm.nih.gov/Structure/cdd/wrpsb.cgi). The tertiary structure of LysSYL was simulated using PHYRE2 Protein Fold Recognition Server (http://www.sbg.bio.ic.ac.uk/phyre2/html/page.cgi?id=index). The structural similar protein sequences compared with LysSYL were downloaded from NCBI. The multiple sequence alignments of these proteins were conducted using ClustalW with default parameters, and the phylogenetic tree was constructed and displayed by MEGA 11 (https://www.megasoftware.net/ mega.php) with the neighbor-joining method [31].

### Cloning, expression, and purification of recombinant LysSYL

The gene encoding the endolysin LysSYL was amplified with polymerase chain reaction (PCR) using a pair of primers (forward: 5΄-GGAATTCcatatgGCAAAAA CGCAGGCCG-3΄ and reverse: 5΄-CCGctcgagTTTAAAAACACCCCATGC-3΄) and cloned into the vector pET-21a(+) between *Nde*I and *Xho*I restriction sites. The recombinant plasmid named pET21a-LysSYL was confirmed sequentially by PCR with external primers (forward: 5΄-CCCGCGAAATTAATACGACTCACTATAGG-3΄ and reverse: 5΄-CAAAAAACCCCTCAAGACCCGTTTAG-3΄), restriction enzyme analysis, and DNA sequencing with PCR products amplified by universal primers T7 (5΄-TAATACGACTCACTATAGGG-3΄)/T7t (5΄-TGCTAGTTATTGCTCAGCGG-3΄).

The correct pET21a-LysSYL was then transformed into competent *E. coli* BL21 (DE3). Furthermore, the positive colony was cultured in LB broth containing 100 µg/mL AMP, shaken at 37 °C (200 rpm) until an OD600 of 0.6, followed by induction with diverse concentrations of isopropyl-β-d-thiogalactoside (IPTG) and incubation at various temperatures for another 16 h. Then, bacterial cells were harvested by centrifugation at 10,000 ×g for 20 min and disrupted with sonication for 30 min at 4 °C. The soluble protein of LysSYL was suspended in the binding buffer (50 mM Na_2_HPO_4_·12H_2_O, 0.5 M NaCl, pH7.5) and purified by His Trap™ FF column (GE Health Bio-Sciences, Sweden) at a rate of 3 mL/min with the elution buffer (50 mM Na_2_HPO_4_·12H_2_O, 0.5 M NaCl, 150 mM imidazole, pH7.5). The purified protein was analyzed by 12% (m/v) sodium dodecyl sulfate polyacrylamide gel electrophoresis (SDS-PAGE). Then, the lipopolysaccharide (LPS) in the recombinant LysSYL solution was removed using endotoxin removal kit (ToxinEraser™, GenScript). After dialysis and enrichment, the concentration of the LysSYL was detected by a bicinchoninic acid assay (Solarbio, Beijing) and stored in − 80 °C. The bactericidal activity of LysSYL was determined by inhibition zone assay.

### Antimicrobial activity of endolysin LysSYL

The lytic activity of LysSYL was examined by inhibition zone assay, turbidity method, or colony forming unit (CFU) reduction assay. For inhibition zone assay, *S. aureus* XN108 was prepared (1 × 10^8^ CFU/mL) and 200 µL was inoculated to a BHI agar plate. Then, about 20 µL of LysSYL (1, 10, and 100 µM, respectively) was separately spotted onto the plate, the same volume of VAN was used as positive control, and PBS used as negative control. The inhibition zones were observed after overnight incubation at 37 °C. For turbidity method, *S. aureus* XN108 and *S. aureus* USA300 were cultivated to the mid-log phage, centrifuged, and washed with PBS. Then, bacterial cells were resuspended in PBS to a final OD600 of 1.0. Subsequently, 180 µL of bacterial suspension was mixed with 20 µL of LysSYL to a final concentration of 50 µg/mL in a 96-well plate, the OD600 value in each well was monitored immediately by a microplate reader (TECAN SPARK20M, Switzerland) at 37 °C for 60 min. The OD600 value was measured every 2 min. The lytic activities of LysSYL against MRSA strains XN108, USA300, and N315, methicillin-sensitive *S. aureus* (MSSA) strains ATCC 25923 and Newman, *P. aeruginosa* strains PAO1 and PA1, *E. coli* strains DH5α and O157:H7, and *A. baumannii* strains ATCC 19606 and AB2 were determined via CFU reduction assay. The strains of interest were each cultivated to the mid-log phage, centrifuged at 6,000 ×g for 10 min, washed twice with PBS, and diluted to 5 × 10^7^ CFU/mL. After that, 80 µL of bacterial suspension was incubated with 20 µL of LysSYL (50 µg/mL in final) at 37 °C for 1 h. For Gram-negative bacteria, 80 µL bacterial culture was pretreated with 2.5 mM ethylene diamine tetraacetic acid (EDTA). Finally, the mixture was serially 10-fold diluted and spotted onto BHI or LB agar plates, which were then cultured overnight at 37 °C. The colonies grown in the plates were calculated. The tests were performed in triplicate. PBS-treated group served as negative control.

### Stability evaluation

Factors affecting LysSYL lytic activity were analyzed using early log phase *S. aureus* XN108 under different conditions. Briefly, LysSYL was incubated at different temperatures (ranging from 4 to 100 °C) for 10 min to evaluate thermostability. After cooled to the ambient temperature, the bacterial suspension was mixed with 50 µg/mL of LysSYL and incubated at 37 °C for 1 h. To determine the influence of pH, NaCl, EDTA and serum on the lytic activity of LysSYL, *S. aureus* XN108 cells were suspended in phosphate buffers with different pH (3–11), phosphate buffer saline (PBS, pH 7.4, 10 mM) containing various concentrations of NaCl (50–500 mM), EDTA (50–500 µM), or mouse serum (12.5%, 25%, 50%, and 100%, v/v). A 20 µL aliquot of LysSYL was tested against 80 µL of *S. aureus* XN108 suspension and incubated at 37 °C for 1 h. The viable bacterial number was calculated by plate dilution method. All experiments were conducted three times, PBS-treated wells served as controls.

### MIC determination

The minimal inhibitory concentration (MIC) of LysSYL against bacteria was determined using the microdilution method. In brief, bacteria were grown in BHI or LB medium at 37 °C overnight with shaking (200 rpm). On the next day, the bacterial culture was inoculated into fresh BHI or LB medium (1:100) and cultured at 37 °C to the logarithmic phase (OD600 = 0.5). Then, 10 µL of the serial dilutions of LysSYL (0.25–256 µg/mL) and 90 µL of bacterial culture (1 × 10^5^ CFU/mL) were added to the 96-well plates. The bacterial mixture was incubated at 37 °C for 18 h. VAN and meropenem (MEM) were used as positive controls. MIC was defined as the minimum concentration with no visible bacterial growth and OD increase after overnight culture.

### Host range and lytic spectrum determination

The host range for phage SYL and lytic spectrum for endolysin LysSYL were determined using a dot dilution assay against 115 staphylococcal strains listed in Additional file 3: Table S1. Briefly, the strains were cultivated to the mid-log phase (OD600 = 0.125–0.25). The plaque forming unit (PFU) of the phage SYL lysate was determined, then 20 µL of phage lysate (~ 10^9^ PFU/mL) and 20 µL of bacterial culture were added to a 1.5 mL centrifuge tube. For the lytic spectrum of LysSYL, the bacterial culture was centrifuged at 6,000 ×g for 10 min, washed with PBS, and 10-fold diluted. Next, 20 µL of LysSYL with a final concentration of 50 µg/mL and 80 µL of bacterial suspension were added to a 1.5 mL centrifuge tube and incubated at 37 °C for 1 h. Finally, the mixtures were 10-fold diluted and 10 µL of each solution was spread onto BHI agar plates. BHI medium and PBS served as negative controls. Bacterial counting was performed based on the colonies after dot dilution assay, and the lytic activity of phage SYL or its LysSYL was assessed by the reduction in bacterial counts: more than 4 log10 units (+++), 2–4 log10 units (++), 0.5–2 log10 units (+), and less than 0.5 log10 units (−). The experiments were conducted three times.

### Live/dead bacterial staining assay

The live or dead status of the tested bacteria was observed through a LIVE/DEAD BacLight staining kit (Invitrogen, CA) and visualized by confocal laser scanning microscopy (CLSM). Bacteria with intact cell membranes were stained green, whereas those with damaged membranes were stained red. Briefly, *S. aureus* USA300 cells in the mid-log phase growth were collected and washed twice with PBS. Then, 800 µL bacterial suspension (1 × 10^8^ CFU/mL) was added to 200 µL of LysSYL with a final concentration of 4×MIC (128 µg/mL), and incubated at 37 °C for 1 h. Subsequently, the mixture was collected, washed once with PBS, and resuspended in PBS. Next, 2 mL suspensions were added into a 35-mm-diameter glass-bottom microwell dish and dyed with a 1:1 mixture of SYTO9 and propidium iodide (PI) in the dark for 15 min. After staining, the bacteria were observed by a Zeiss LSM880 confocal microscope (Carl Zeiss, Germany). Moreover, the real-time observation of the bactericidal process was also captured by CLSM.

### Electron microscope observation of bacteria

The morphological changes of *S. aureus* cells after treatment with endolysin LysSYL were observed with electron microscopes. Briefly, *S. aureus* USA300 cells (1 × 10^8^ CFU/mL) were incubated with 4×MIC LysSYL at 37 °C for 1 h, then the mixture was centrifuged at 6,000 ×g for 10 min, washed once with PBS, and fixed in 2.5% (v/v) glutaraldehyde at 4 °C overnight. For scan electron microscopy (SEM), bacterial cells were washed with PBS, dehydrated with a graded ethanol series, and dried by CO_2_. Next, the samples were sputtered with platinum coating and observed with a JSM-IT700HR SEM (JEOL, Japan). For TEM assay, bacterial cells were processed as mentioned above. The thin sections were prepared and stained with uranyl acetate and lead citrate, then observed under a JEM-1400-FLASH TEM (JEOL, Japan).

### Biofilm elimination assays

#### Crystal violet staining assay for biofilms

The antibiofilm ability of LysSYL was determined by crystal violet staining assay as described previously [32]. In brief, 100 µL of *S. aureus* USA300 culture (10^8^ CFU/mL) was inoculated into 96-well plates supplied with 100 µL of BHIg (BHI added with 1% (m/v) glucose) medium. The plates were incubated in static at 37 °C for 24 h or 72 h to allow biofilm formation. Once the biofilm was formed, the wells were washed twice with PBS to remove planktonic cells, then treated with 200 µL of LysSYL (1/8×MIC–2×MIC) or VAN (4×MIC–64×MIC) at 37 °C for 1 h and 5 h for the 24 h- and 72 h-established biofilms, respectively. Next, the wells were washed gently with PBS, dried and fixed with 2.5% (v/v) glutaric dialdehyde for 90 min. After washing twice with PBS, 200 µL of 0.1% (m/v) crystal violet was added to each well and stained at room temperature for 10 min. Subsequently, the wells were washed with PBS, dissolved in 200 µL 33% (v/v) glacial acetic acid for 30 min, and read OD595 values with a microplate reader (Thermo Scientific™ Multiskan™ GO, USA). PBS treated wells used as negative controls.

#### Mixed biofilm disruption

Dual-species biofilms of *S. aureus* and *A. baumannii* were established as described [33, 34]. *S. aureus* USA300 and *A. baumannii* ATCC 19606 or AB2 were cultured and bacterial numbers were counted. Then, *S. aureus* suspension was mixed with *A. baumannii* in a bacterial number ratios of 1:1, 10:1, and 100:1, respectively. The mixtures were dispensed into 96-well plates, which were incubated at 37 °C for 24 h to allow biofilm formation. Mono-species *A. baumannii* biofilms were generated by the addition of 100 µL of bacterial culture (10^8^ CFU/mL) and 100 µL of LBg (LB added with 1% (m/v) glucose) medium into 96-well plates. After incubation at 37 °C for 24 h, the wells were washed twice with PBS and treated with 200 µL of LysSYL (1/8×MIC–2×MIC) for 1 h. Next, the wells were washed gently with PBS, dried and fixed with 2.5% (v/v) glutaric dialdehyde for 90 min. After washing twice with PBS, the wells were stained with crystal violet assay. VAN and MEM were used as positive controls. PBS used as negative control.

#### Biofilm bacterial counting

To assess the biofilm-killing efficacy of LysSYL, the viable bacterial number in the 24 h-established biofilms was calculated by plate dilution assay. Briefly, 100 µL of *S. aureus* USA300 suspension (2 × 10^8^ CFU/mL) supplied with 100 µL of BHIg medium were added into 96-well plates and incubated at 37 °C for 24 h. Next, the wells were washed with PBS and treated with 200 µL of LysSYL (1/8×MIC–2×MIC) or VAN (4×MIC–64×MIC) for 1 h. Then, the wells were washed and resuspended in PBS, 10-times diluted, and spotted onto BHI agar plates. After culture at 37 °C overnight, the colonies were counted.

#### Observation of biofilms by CLSM

The effect of LysSYL on the biofilms was assessed using a LIVE/DEAD BacLight staining kit and visualized using CLSM. Briefly, 1 mL of *S. aureus* USA300 culture (2 × 10^8^ CFU/mL) and 1 mL of fresh BHIg medium were added into a 35-mm-diameter glass-bottom microwell dish, then the dishes were incubated at 37 °C for 24 h or 72 h. After incubation, the biofilms were treated with 1×MIC LysSYL (32 µg/mL) for 1 and 5 h, respectively. After washing with PBS, the biofilms were dyed with a 1:1 mixture of SYTO9 and PI in the dark for 15 min. Finally, the biofilms were visualized using a Zeiss LSM880 confocal microscope. 32×MIC VAN (32 µg/mL) treatment served as positive control, and PBS treated wells were used as negative controls.

#### Microscopic analysis of biofilms

*S. aureus* USA300 biofilms were prepared as described above. The 24 h-biofilms were treated with 1×MIC LysSYL for 1 h, and the 72 h-biofilms were treated with 1× MIC LysSYL for 5 h. Then, the wells were immobilized with 2.5% (v/v) glutaric dialdehyde, dehydrated with different concentrations of ethanol, air-dried, and observed under an S-3400 N П electron microscope (Hitachi, Japan). 32×MIC VAN treatment served as positive control, and PBS treated well used as negative control.

### Killing activity of LysSYL against *S. aureus* persisters

The lytic activity of LysSYL against *S. aureus* persisters was determined as described previously [35]. Briefly, overnight culture of *S. aureus* USA300 was inoculated into fresh BHI medium and grown to an OD600 of 0.5 at 37 °C with shaking. Next, 4.8 mL of bacterial culture was aliquoted into test tubes, and 200 µL of VAN or cefuroxime (CEF) was added to achieve concentrations corresponding to 100×MIC. Then, the tubes were incubated at 37 °C with shaking, and 300 µL aliquots were taken at 2, 4, 8, and 24 h time points for CFU quantification. A rapid decrease in the CFU, followed by stable CFU values up to 24 h, indicated the presence of antibiotic-tolerant persisters. The persisters were treated with LysSYL (4×MIC) for 1 or 6 h. The aliquots at different time points were pelleted, washed, diluted, and plated onto BHI agar plates. The colonies were counted after culture.

### Endolysin LysSYL toxicity assays

Normal BALB/c mice aged 6–8 weeks were randomly divided into three groups (*n* = 5 for each), and intraperitoneally challenged with LysSYL (50 mg/kg). VAN (5 mg/kg) and PBS served as controls. The mice were weighed daily and observed up to 14 days. Finally, the mice were euthanized, and organs were collected for observation of gross lesion changes and pathological variations after hematoxylin and eosin (H&E) staining as described [36].

### Treatment efficacy of LysSYL for MRSA infection

A mouse peritonitis model caused by *S. aureus* USA300 was generated as described previously [32, 36]. The BALB/c mice (*n* = 6 per group) were intraperitoneally injected 200 µL PBS containing different concentrations of *S. aureus* USA300 (1 × 10^8^, 2.5 × 10^8^, 5 × 10^8^, and 1 × 10^9^ CFU/mL) to determine the minimal lethal dose (MLD) that caused 100% mortality within one day. All animal experiments were approved by the Laboratory Animal Welfare and Ethics Committee of Army Medical University (SYXK-PLA-20,120,031). Euthanasia of the experimental animals was performed by cervical dislocation.

Next, the mice were inoculated intraperitoneally with MLD as the challenge dose. The infected mice were intraperitoneally treated with 200 µL of LysSYL (12.5, 25 or 50 mg/kg) diluted in PBS after 1 h infection with MLD of *S. aureus* USA300 (5 × 10^8^ CFU/mL, 200 µL). Survival of mice was recorded daily for 7 days. VAN (1.25, 2.5 or 5 mg/kg) treated mice served as positive controls, and PBS treatment used as negative control.

In addition, the infected mice were intraperitoneally treated with LysSYL (50 mg/kg) and VAN (5 mg/kg) 1 h after infection. Then, the mouse organs (liver, spleen, lungs, and kidneys) were obtained at 24 h post-treatment, weighed, and homogenized for CFU counting. For histopathological analysis, mouse organs were collected at 72 h post-treatment and fixed in 4% (v/v) formalin. After paraffin embedding, the fixed tissues were sectioned, stained with H&E, and examined by a light microscope (Olympus BX53, Japan).

### Statistical analysis

All data were analyzed with GraphPad Prism 9.5 and presented as mean ± standard deviation (SD). Statistical analysis was performed by one-way or two-way analysis of variance (ANOVA). *P* < 0.05 was considered statistically significant.

## Results

### Phage isolation and endolysin characterization

**Fig. 1:**
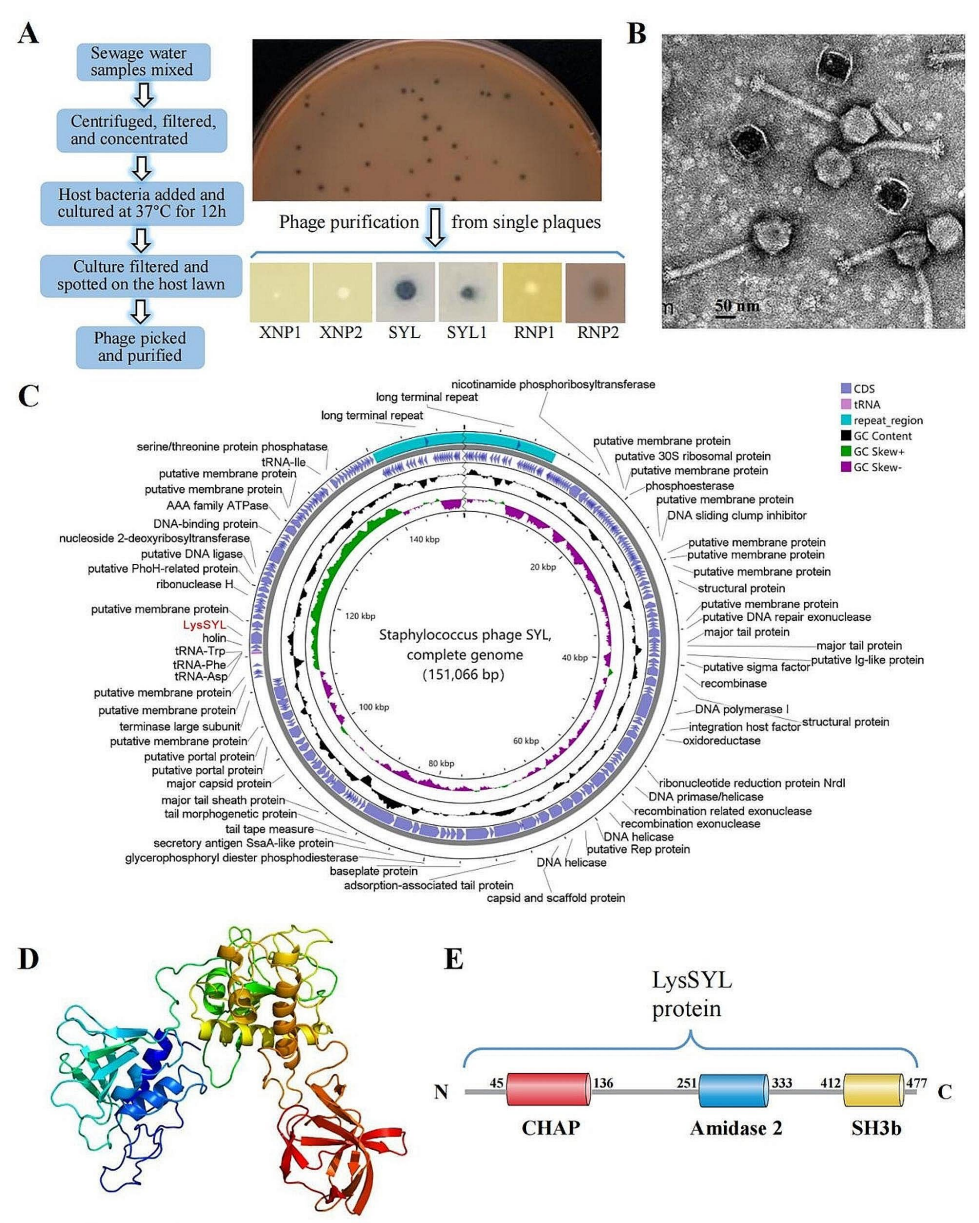
Isolation and identification of *S. aureus* phage SYL. **A** Phages against MRSA XN108 obtained after an enrichment procedure. A schematic diagram showing the enrichment procedure (left panel). Phages with various lytic phenotypes against MRSA XN108 were purified and named (right panel). **B** Representative transmission electron micrographs of the phage SYL. **C** Genomic map of *S. aureus* phage SYL showing coding sequences (CDSs), tRNA, repeat region, GC content, GC skew^+^, and GC skew^−^ by schematic diagrams. **D** Predicted tertiary structure model of endolysin LysSYL. **E** Schematic illustration of structure of endolysin LysSYL.

A total of 20 sewage water samples obtained were combined, centrifugated, and enriched using tangential flow filtration. Subsequently, an ST239 MRSA strain XN108 with a VAN MIC of 12 µg/mL (VISA) was used as the host cell to isolate phages from the mixed-sample. The resulting phages displayed diverse lytic phenotypes. Among them, a phage referred to as SYL was obtained. SYL could form clear and transparent plaques with approximately 3 mm in diameter on double layer agar plates after repeated purification (Fig. 1A). Phage particles were then prepared and examined through TEM observation. The results showed that SYL had a long tail (Fig. 1B). A complete genome sequencing of phage SYL was performed (GenBank accession number: OP235318.1), which revealed that the phage carried a circular genomic DNA of 151,066 bp with a G + C content of 30.2% (Fig. 1C). The phylogenetic analysis showed that there was a specific evolutionary distance between SYL and other phage members within the *Kayvirus* genus, *Herelleviridae* phage family (Additional file 3: Fig. S1). Moreover, phage SYL possessed 250 putative coding sequences (CDSs), which only a small proportion of them encoding proteins

with known functions (23.2%, 58/250) (Fig. 1C). Among the predicted functional proteins, one particular molecule (protein ID: UVD37129.1) was identified as an N-acetylmuramoyl-L-alanine amidase, which is a key enzymatic activity of phage endolysin [23]. Thus, we designated this protein as the phage endolysin LysSYL (Fig. 1C). LysSYL consists of 495 amino acids and has a theoretical molecular weight of 54.7 kDa. LysSYL exhibits a three-domain structure (Fig. 1D), and functionally contains two CD domains (CHAP and amidase 2) and one bacterial Src homology 3 (SH3b) that serves as a CBD domain (Fig. 1E). Phylogenetic analysis revealed that LysSYL was distantly related to the previously characterized structural similar endolysins from phages Twort, phi11, 2638 A, phiSH2, and phiWMY (Additional file 3: Fig. S2).

### LysSYL is stable under various physical and chemical conditions

**Fig. 2:**
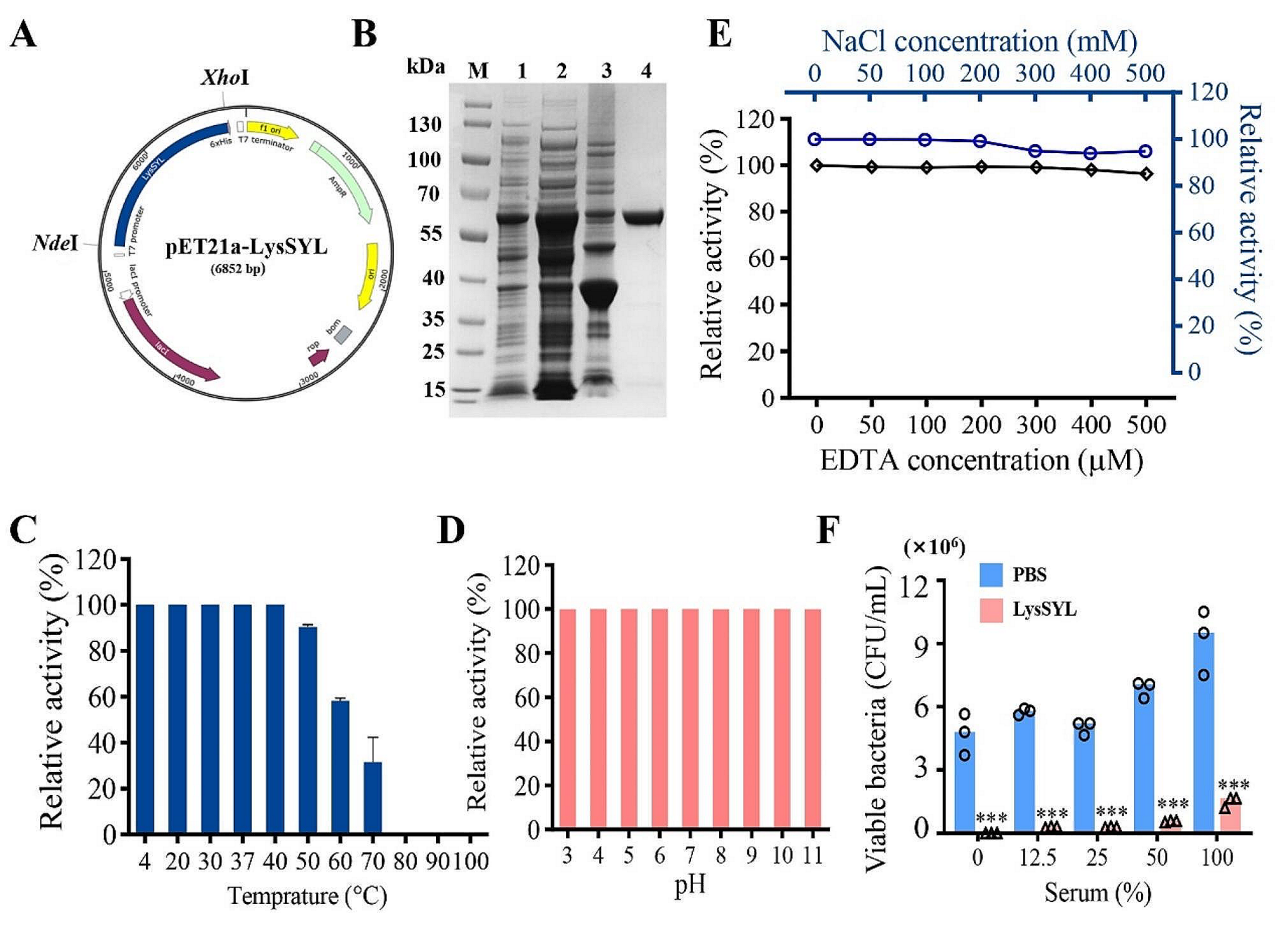
Preparation and stability of the recombinant endolysin LysSYL. **A** Schematic diagram showing the recombinant plasmid pET21a-LysSYL. **B** SDS-PAGE analysis of the purified endolysin LysSYL. Lane 1, *E. coli* BL21/pET21a-LysSYL. Lane 2, Supernatant of the IPTG-induced bacteria after sonication. Lane 3, Pellet of the IPTG-induced bacteria after sonication. Lane 4, endolysin LysSYL after purification with Ni-NTA column. M, protein marker. The sizes of the marker are indicated on the left. **C** Stability of the recombinant endolysin LysSYL in diverse temperature. The activity was shown relative to that of LysSYL at 4 °C. LysSYL stability in various (**D**) pH, and (**E**) EDTA (black line) and NaCl (blue line). **F** Bactericidal activity of LysSYL in different concentrations of mouse sera. PBS served as control. Viable bacteria were counted with plate dilution assay. The experiment was conducted three times. Data were presented as mean ± standard derivation (SD). Significance was calculated by two-way ANOVA between LysSYL treatment and PBS control. ***, *P* < 0.001

The gene encoding LysSYL was cloned to produce the recombinant plasmid pET21a-LysSYL for generating the recombinant endolysin (Fig. 2A; Additional file 3: Fig. S3). The expression of LysSYL in *E. coli* BL21 was optimized with 0.5 mM of IPTG induction for 16 h at 23 °C (Additional file 3: Fig. S4), and the target proteins were purified from soluble bacterial lysates (Fig. 2B).

The inhibition zone assay was conducted to assess the bactericidal activity of the recombinant LysSYL. The results showed that LPS-free LysSYL could produce clear inhibition zones when cultured with MRSA XN108 (Additional file 3: Fig. S5). The stability of the recombinant endolysin is critical for its application [26]. LysSYL exhibited good thermal stability by remaining active at temperatures ranging from 4 to 50 °C (Fig. 2C). However, its bactericidal activity gradually decreased at temperatures exceeding 50 °C and was completely abolished at 80 °C. Moreover, LysSYL maintained consistent activity across a pH range of 3–11 (Fig. 2D) and in the presence of NaCl concentrations in the range of 0–500 mM and the EDTA concentrations in the range of 0–500 µM (Fig. 2E). Mammalian serum is a strict physical condition encountered by exogeneous active substances in vivo [26]. Our data demonstrated that LysSYL retained its lytic activity in distinct concentrations of mouse sera. Notably, 83.6% of MRSA XN108 cells was killed after treatment with LysSYL in 100% sera (Fig. 2F).

### LysSYL kills a wider range of bacteria than its parental phage

**Fig. 3:**
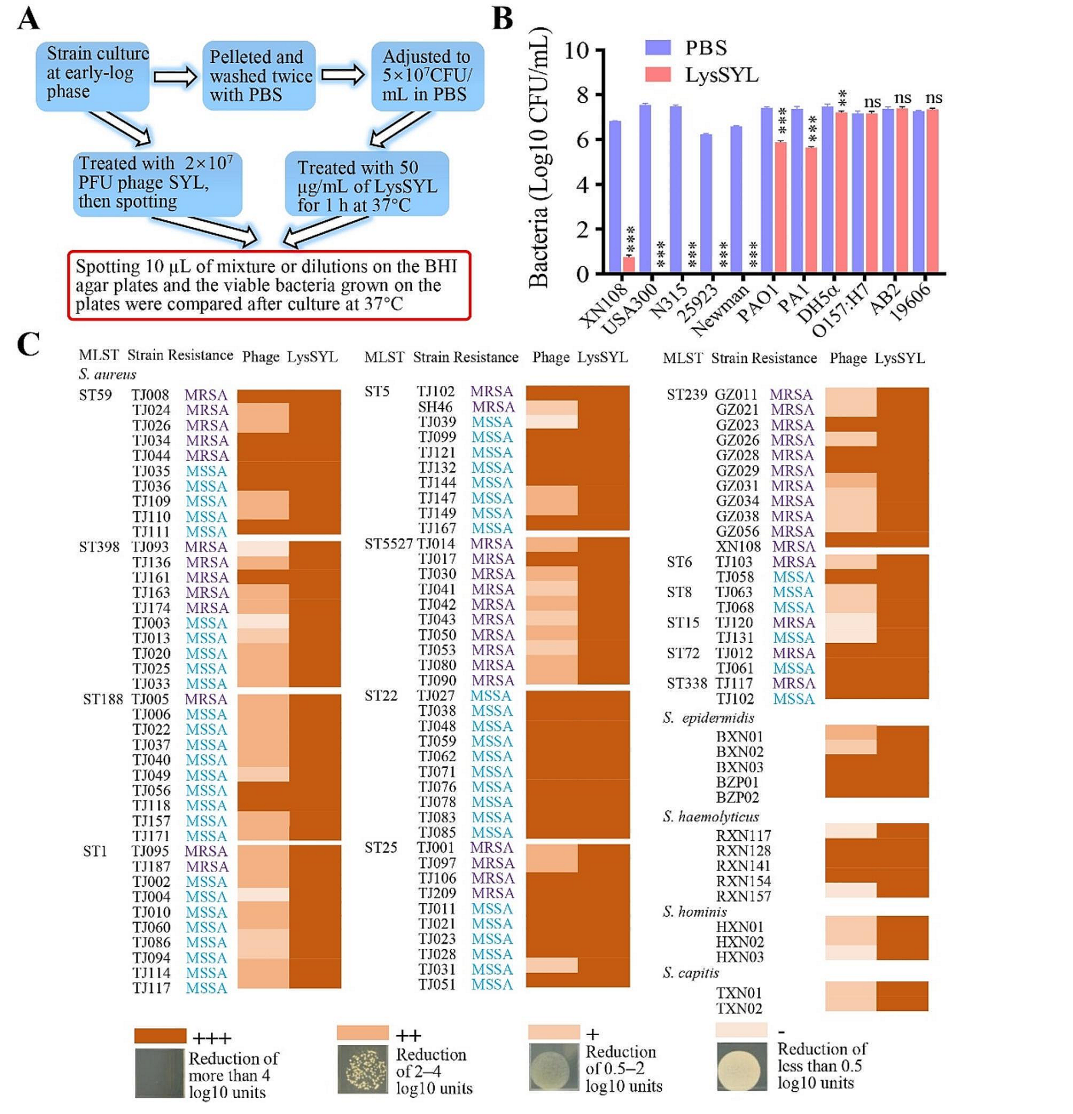
Antimicrobial activity of endolysin LysSYL and phage SYL. **A** Schematic diagram showing the procedure of host range determination assay. **B** CFU reduction assay for testing lytic activity of LysSYL against MRSA (XN108, USA300, and N315), MSSA (25923 and Newman), *P. aeruginosa* (PAO1 and PA1), *E. coli* (DH5α and O157), and *A. baumannii* (AB2 and 19606). The experiment was repeated three times. Data were presented as mean ± SD. Significance was calculated by two-way ANOVA between LysSYL treatment and PBS control, ns representing no significance, and *** *P* < 0.001. **C** Comparison of lytic spectrum of the phage SYL and endolysin LysSYL. Sequence types (STs) and resistance phenotypes of *S. aureus* strains were presented. Lytic activity was gradually divided into four grades (+++, ++, +, and −) with phenotypes and log10 unit reductions as indicated.

The bactericidal activity of LysSYL against various bacterial strains was assessed, including MRSA strains XN108, N315, and USA300; MSSA strains ATCC 25923 and Newman; *P. aeruginosa* strains PAO1 and PA1 (a carbapenem-resistant strain); *E. coli* strains DH5α and O157; and *A. baumannii* strains ATCC 19606 and AB2 (carbapenem-resistant). This determination was conducted using a CFU reduction assay (Fig. 3A). Bacterial cells in the mid-log phase growth (OD600 = 0.5) were collected and washed with PBS. Then, 80 µL of cell suspension (1 × 10^7^ CFU/mL) of each strain was incubated with 20 µL of LysSYL at a final concentration of 50 µg/mL at 37 °C for 1 h. Bacterial colonies were counted by the plate dilution method, and the results demonstrated that LysSYL exhibited strong bactericidal activity against all tested *S. aureus* strains, which resulted in a notable reduction of over 6 log10 units (Fig. 3B). Furthermore, LysSYL significantly lysed *P. aeruginosa* PAO1 and PA1 (*P* < 0.001) and *E. coli* DH5α (*P* < 0.01). However, its activities against *E. coli* O157:H7 and *A*. *baumannii* strains were less effective. However, no activity was observed in killing *P. aeruginosa*, *E. coli*, and *A*. *baumannii* strains when using the parent phage SYL (Additional file 3: Fig. S6). Therefore, LysSYL possessed a wider lytic spectrum than its parent phage SYL.

To further compare the host range of phage SYL and the lytic spectrum of endolysin LysSYL, a CFU reduction assay was performed using representative clinical isolates of *S. epidermidis*, *S. haemolyticus*, *S. hominis*, and *S. capitis*, as well as major prevalent clones of *S. aureus* isolated from Chongqing, Tianjin, and Guangzhou (Additional file 3: Table S1). As shown in Fig. 3C, endolysin LysSYL exhibited a high bactericidal activity (+++) against all 115 staphylococcal isolates, whereas phage SYL effectively (+++) killed only 41.7% (48/115) of the strains, with 9 isolates proving completely resistant to phage treatment (−). *S. aureus* ST22, ST72, and ST338 clonal strains appeared more susceptible to phage SYL inactivation, while ST398, ST188, ST1, ST5527, ST8, and ST15 *S. aureus*, *S. hominis*, and *S. capitis* isolates presented relative resistance to phage killing. These data strongly indicate that endolysin LysSYL exhibits a superior capacity to lyse staphylococcal pathogens when compared with its parent phage SYL.

### LysSYL rapidly disrupts *S. aureus* cells

LysSYL exhibited bactericidal activity against *S. aureus* XN108 in a dose-dependent manner (Additional file 3: Fig. S7A). In a turbidity examination, the OD600 values of *S. aureus* XN108 and USA300 reduced rapidly after exposure to 50 µg/mL of LysSYL. Notably, LysSYL rapidly reduced the OD600 value of the *S. aureus* USA300 culture from 1.09 to 0.48 within 2 min. By contrast, a relatively gradual reduction in the OD600 curve was observed in the case of *S. aureus* XN108 treated with LysSYL (Additional file 3: Fig. S7B). These findings suggest that different *S. aureus* strains exhibit various susceptibilities to LysSYL inactivation, which was confirmed by the CFU reduction assay (Fig. 3B).

**Fig. 4:**
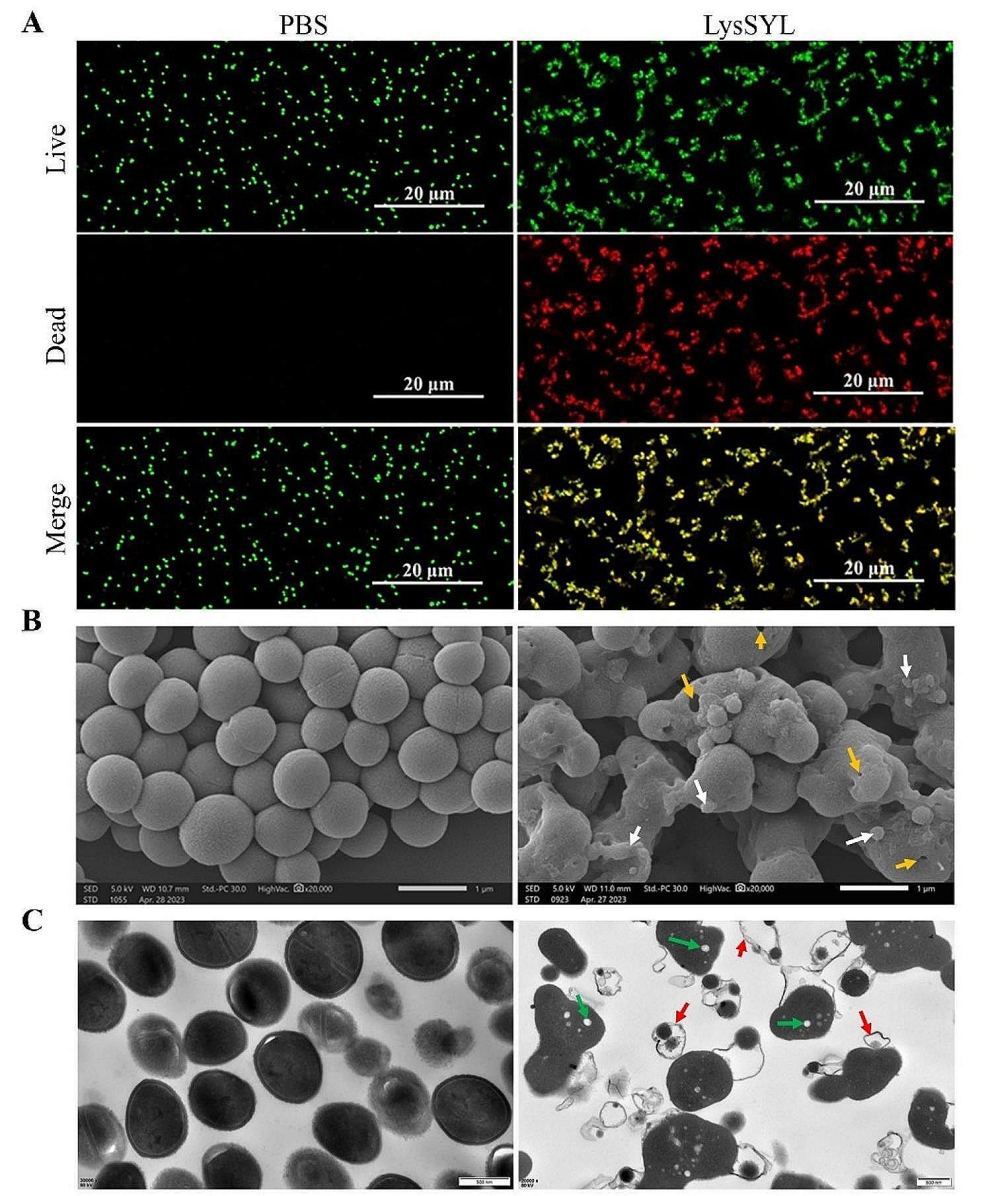
LysSYL rapidly killed *S. aureus* cells. **A** Fluorescent imaging of live/dead bacterial staining. *S. aureus* USA300 cells were treated with 4×MIC of LysSYL at 37 °C for 1 h. Green fluorescence showed living bacteria and red fluorescence indicated dead bacteria only. PBS treatment served as negative control. **B** SEM observation of *S. aureus* USA300 cells after treatment with 4×MIC of LysSYL for 1 h. PBS treatment served as control. The holes formed after LysSYL treatment were indicated by yellow arrows, and the bacterial effluents were showed by white arrows. White bars represent 1 μm. **C** TEM observation of *S. aureus* USA300 cells treated with 4×MIC of LysSYL. PBS treatment served as control. Low electron density regions (green arrows) increased and many cellular debris or bacterial ghost cells (red arrows) exhibited. Bars represent 500 nm.

The MIC of LysSYL against diverse *S. aureus* strains was determined, and the results are shown in Additional file 3: Table S2. Compared with VAN (MIC = 8.08 µM), the activity of LysSYL against *S. aureus* XN108 (MIC = 2.34 µM) increased 3.5 times. Live and dead bacterial observations indicated that nearly all *S. aureus* USA300 cells were killed after exposure to 4×MIC of LysSYL at 37 °C for 1 h (Fig. 4A). Dynamic observations using a confocal microscope revealed that the fluorescence intensities of PI for dead bacterial staining in *S. aureus* USA300 treated with LysSYL were markedly enhanced within 10 min (Additional file 1: Movie S1) when compared with those in bacteria after PBS treatment (Additional file 2: Movie S2). These results demonstrate that LysSYL can rapidly lyse *S. aureus* cells.

Bacterial cell damage was further examined by SEM and TEM. *S. aureus* treated with PBS presented an intact cellular morphology and a smooth surface. On the contrary, evident cell perforation and deformation were observed in *S. aureus* USA300 cells treated with endolysin LysSYL (Fig. 4B). TEM revealed that the cells treated with PBS maintained their well-defined cell envelope structures (Fig. 4C). By contrast, LysSYL-treated staphylococcal cells exhibited in a large population of bacterial ghosts devoid of cytoplasmic materials. Even in the case of bacterial cells with intact envelope structures, they exhibited the formation of dense electron densities and empty regions following treatment with LysSYL (Fig. 4C). These findings indicate that *S. aureus* cells can be completely lysed after 1 h of endolysin treatment, which primarily results in bacterial debris.

**LysSYL efficiently disperses*S. aureus*mono-species biofilms**.

**Fig. 5:**
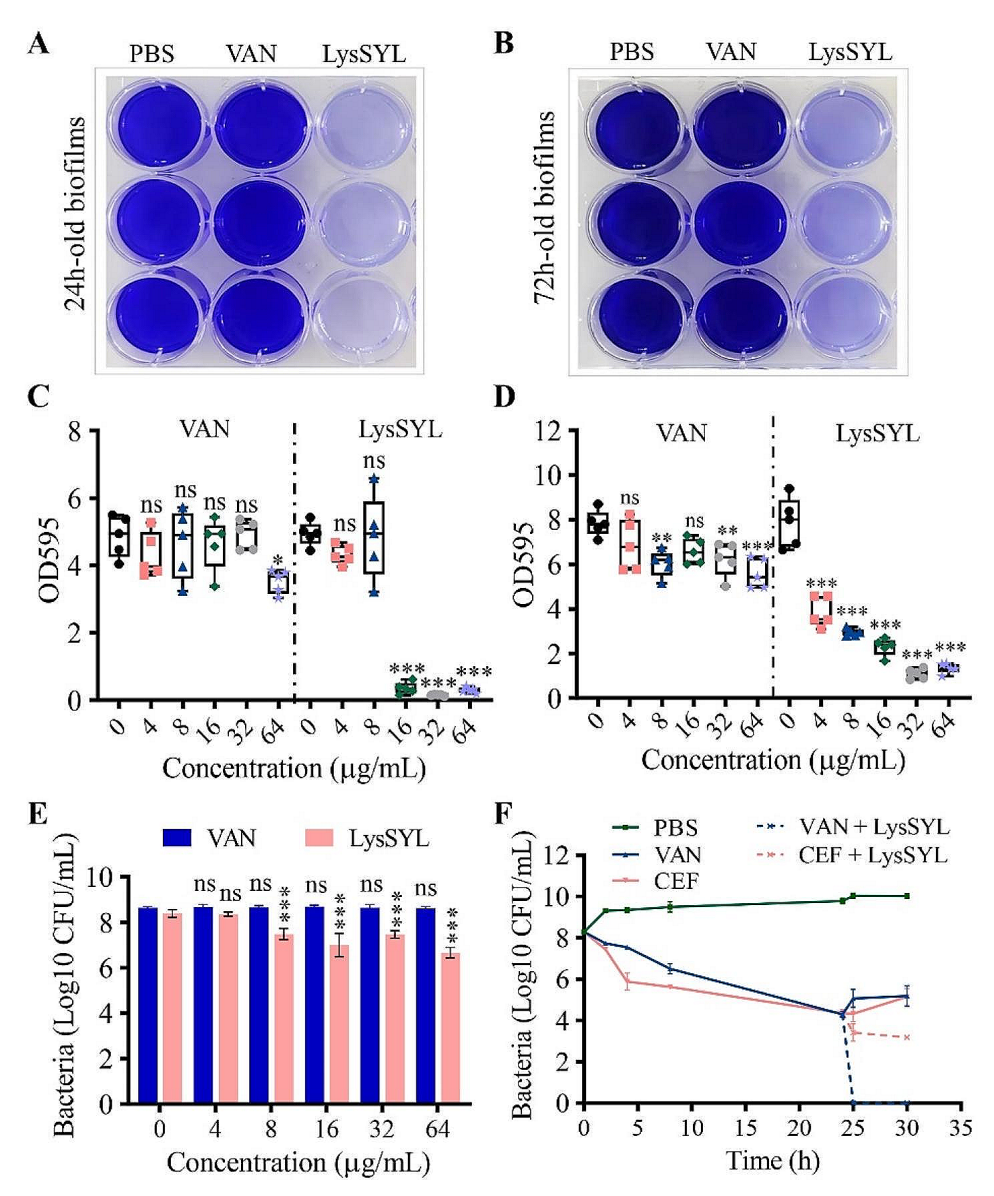
Disruption efficacy of endolysin LysSYL against mono-species biofilms of *S. aureus* and its persisters. Representative images of (**A**) 24 h and (**B**) 72 h *S. aureus* USA300 biofilms treated with 1× MIC LysSYL for 1 and 5 h, respectively. 32×MIC VAN treatment served as positive control, and PBS used as negative control. Eradication of (**C**) 24 h- and (**D**) 72 h-old *S. aureus* mono-species biofilms with various concentrations of LysSYL for 1 and 5 h, respectively. The biofilm mass in each well was determined by crystal violet assay. VAN and PBS were used as positive and negative controls, respectively. The experiment was repeated three times. Data were expressed as mean ± SD. The statistical analysis was measured by one-way ANOVA. *P < 0.05, **P < 0.01, ***P < 0.001, and ns indicates no significance. **E** Bactericidal activity of LysSYL against *S. aureus* cells in the biofilms. The number of viable bacteria in the 24 h-old biofilms were counted after treatment with various concentrations of LysSYL for 1 h. VAN served as positive control. The experiment was repeated three times. Data were expressed as mean ± SD. The statistical analysis was measured by two-way ANOVA. ***P < 0.001, and ns indicates no significance. **F** Bactericidal activity of LysSYL against *S. aureus* persisters. Time-kill kinetic graph showing *S. aureus* USA300 cells initially killed with 100×MIC VAN or 100×MIC CEF to achieve a constant number of persisters. Then 4×MIC LysSYL was added at 24 h to remove antibiotic-tolerant persisters. Experiments were repeated three times. Data were expressed as mean ± SD.

*S. aureus* is known for its multidrug-resistant nature, which is primarily due to its ability to form biofilms that are notoriously difficult to remove [37]. The antibiofilm activity of endolysin LysSYL, which is known for its rapid bactericidal activity against *S. aureus*, was assessed with a crystal violet assay, as described previously [38]. The results showed that 1×MIC of LysSYL (32 µg/mL) could significantly remove 24 h- and 72 h-old biofilms established by MRSA USA300, whereas 32×MIC of VAN had a less pronounced effect (32 µg/mL, Fig. 5A, B). Treatment with 16 µg/mL of LysSYL (1/2×MIC) at 37 °C for 1 h could eradicate more than 90% of 24 h-old MRSA USA300 biofilms, while treatment with 32 µg/mL of VAN resulted in biofilm biomass comparable to that of the PBS control (Fig. 5C). For the eradication of 72 h-old *S. aureus* biofilms, LysSYL exhibited a concentration-dependent antibiofilm activity. Treatment with 4 µg/mL of LysSYL (1/8×MIC) for 5 h significantly decreased the biofilm biomass (*P* < 0.001, Fig. 5D). On the contrary, the effect of VAN on the removal of *S. aureus* biofilms was relatively weak, and treatment with 8 µg/mL of VAN (8×MIC) resulted in only a marginal reduction of 1.0 OD595 value (Fig. 5D).

**Fig. 6:**
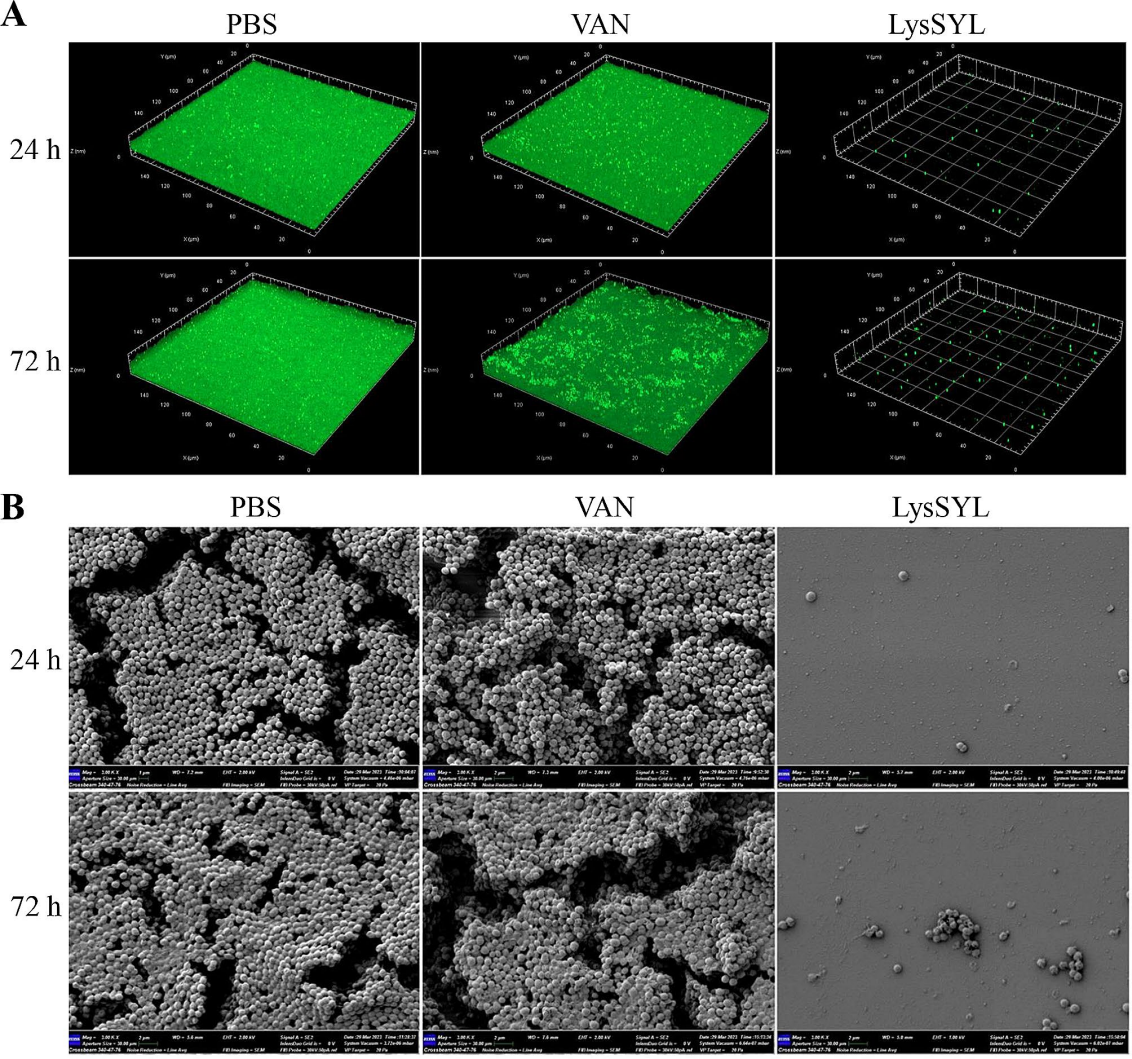
Observation of *S. aureus* biofilms after treatment with 1×MIC of endolysin LysSYL. **A** CLSM images of *S. aureus* mono-species biofilms. The 24 h- and 72 h-old biofilms of *S. aureus* USA300 were treated with 1×MIC LysSYL for 1 and 5 h, respectively. 32×MIC VAN served as positive control, and PBS was used as negative control. The biofilm images were taken after Live/Dead staining. **B** SEM images of *S. aureus* biofilms. The mono-species biofilms of *S. aureus* USA300 (24 and 72 h) after treatment with 1×MIC LysSYL for 1 and 5 h, respectively. 32×MIC VAN served as positive control, and PBS was used as negative control. The bars represent 2 μm

CLSM revealed that 24 h- and 72 h-old MRSA USA300 biofilms could be effectively removed under treatment with 32 µg/mL of LysSYL for 1 and 5 h, respectively (Fig. 6A). By contrast, treatment with 32 µg/mL of VAN resulted in a large proportion of the biofilm remaining compared with the PBS control. Similar findings were observed during SEM examination (Fig. 6B). These data confirm that endolysin LysSYL is highly effectively in removing *S. aureus* biofilms.

The number of viable *S. aureus* USA300 cells in the 24 h-old biofilms gradually decreased by 0.91–1.73 log10 units with the increasing concentrations of LysSYL treatment (4–64 µg/mL) for 1 h, whereas the same concentrations of VAN displayed no activity against biofilm-embedded *S. aureus* (Fig. 5E). In addition, the bactericidal activity of LysSYL against biofilm-embedded *S. aureus* persisters was investigated. As shown in Fig. 5F, the time-killing kinetic graph revealed that the treatment of *S. aureus* USA300 cells with 100×MIC of VAN or 100×MIC of CEF for 24 h led to a reduction of about 5 log10 units. However, the remaining survival bacteria were not easy eradicated completely by either of the two antibiotics and exhibited some growth. At 24 h post-VAN treatment, 4×MIC of LysSYL was added and treated for another 1 h, which led to the complete elimination of VAN-tolerant persisters, with no bacterial growth observed after 6 h of exposure to LysSYL (Fig. 5F). However, treatment with the combination of 100×MIC CEF and 4×MIC LysSYL for 6 h resulted in the reduction of only 1.17 log10 units of CEF-tolerant persisters. These results demonstrate that LysSYL can effectively eradicate bacterial persisters, and the combination of LysSYL and VAN is even more powerful in killing persistent *S. aureus* cells.

### LysSYL eliminates mixed-species biofilms associated with *S. aureus*

**Fig. 7:**
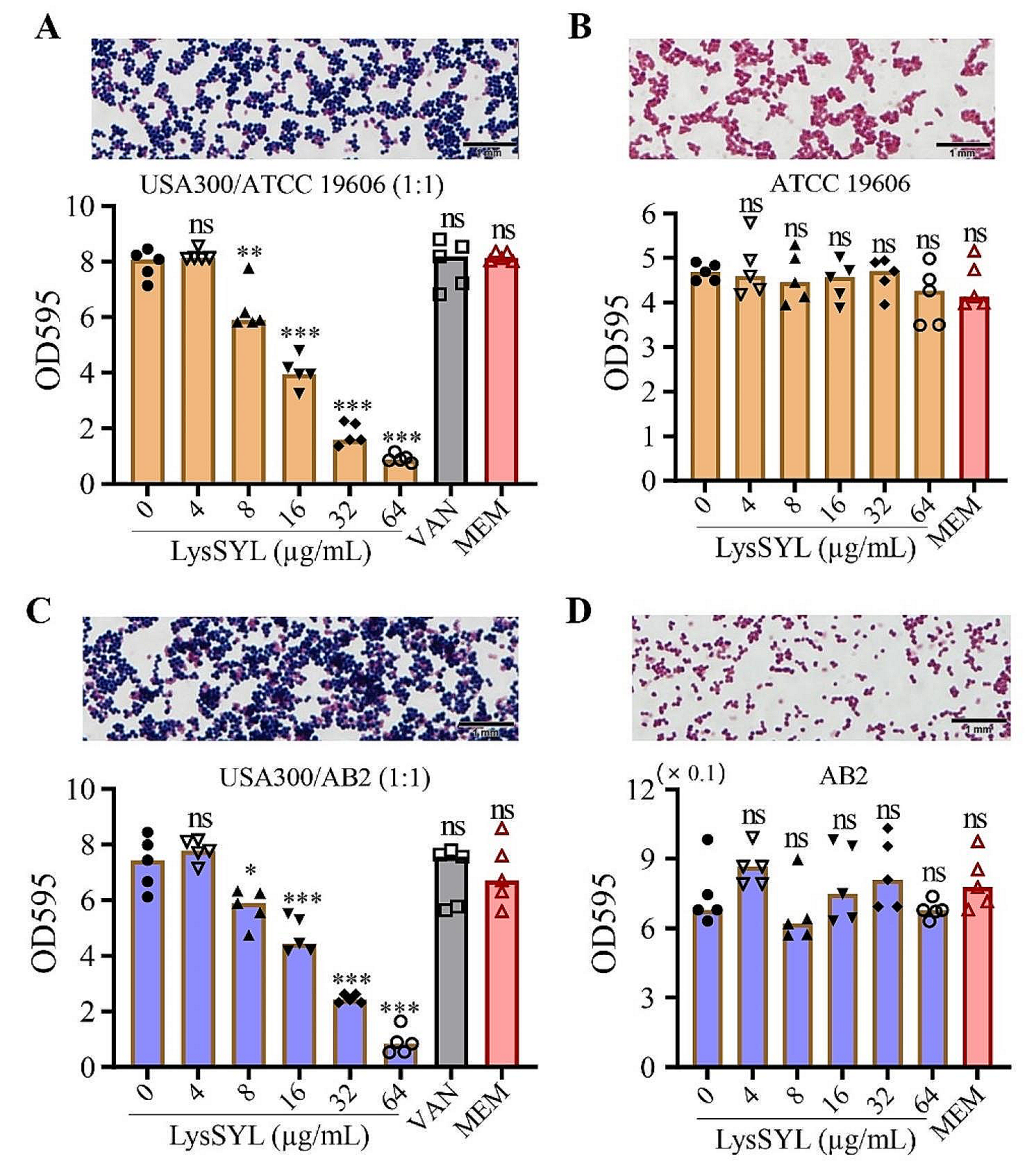
LysSYL disrupted mixed-species biofilms associated with *S. aureus*. Disruption of dual-species biofilms formed by (**A**) *S. aureus* USA300 /*A. baumannii* ATCC 19606 and (**C**) *S. aureus* USA300/*A. baumannii* ATCC AB2 inoculated in a ratio of 1:1, respectively. The bacteria in the mixed-species biofilms were confirmed by Gram staining (up panels). Untreated bacterial biofilms were subjected to Gram-staining. The 24 h-old mixed biofilms were treated for 1 h with various concentrations of LysSYL as indicated, VAN (64 µg/mL) and MEM (128 µg/mL for ATCC 19606 and 8.192 mg/mL for AB2) served as controls (bottom panels). Disruption of single-species biofilms of (**B**) *A. baumannii* ATCC 19606 and (**D**) *A. baumannii* AB2. Bacterial cells in the biofilms were shown by Gram staining (up panels). The 24 h biofilms were treated for 1 h with various concentrations of LysSYL, MEM served as control (bottom panels). The biofilm mass in each well was determined by crystal violet assay. Data were expressed as mean ± SD. The analyses were measured by one-way ANOVA. *P < 0.05, **P < 0.01, ***P < 0.001, and ns showed no significance relative to 0 µg/mL of LysSYL treatment

*S. aureus* is a notorious pathogen involved in polymicrobial biofilm infections, and its co-infections with *A. baumannii* or *P. aeruginosa* have been frequently reported [13]. Mixed-species biofilms of *S. aureus* and *A. baumannii* in diverse inoculation ratios (1:1, 10:1, and 100:1) were generated in 96-well plates for 24 h. LysSYL demonstrated the ability to disrupt dual-species biofilms of *S. aureus* USA300 and *A. baumannii* ATCC 19606 (1:1) in a dose-dependent manner, whereas 64×MIC of VAN (against *S. aureus* USA300) and MEM (against *A. baumannii* ATCC 19606) did not exhibit biofilm elimination effect (Fig. 7A). Moreover, 16 µg/mL of LysSYL significantly eliminated biofilms formed by *S. aureus* USA300 and *A. baumannii* ATCC 19606 (10:1 and 100:1) (Additional file 3: Fig. S8A). However, LysSYL was ineffective against the *A. baumannii* ATCC 19606 single-species biofilms (Fig. 7B).

In addition, a carbapenem-resistant *A. baumannii*, AB2 (Additional file 3: Table S2), was used to establish mixed-species biofilms with *S. aureus* USA300, and similar results were achieved (Fig. 7C, D, and Additional file 3: Fig. S8B). These results strongly indicate that endolysin LysSYL is highly effective in disrupting mixed-species biofilms associated with *S. aureus*, which indicates its promising potential for clinical application in the treatment of polymicrobial infections.

### LysSYL protects mice from lethal *S. aureus* infections

To evaluate the therapeutic potential of LysSYL in vivo, its safety was evaluated using BALB/c mice through intraperitoneal injection. The body weights of mice challenged with LysSYL (50 mg/kg) and VAN (5 mg/kg) were comparable to those of the PBS control group within 14 days of monitoring (Additional file 3: Fig. S9A). At 14 days after injection, no gross lesions and pathologic changes were observed in the liver, spleen, lungs, and kidneys of the mice (Additional file 3: Fig. S9B, C), which indicates that the administration of tested LysSYL was safe.

**Fig. 8:**
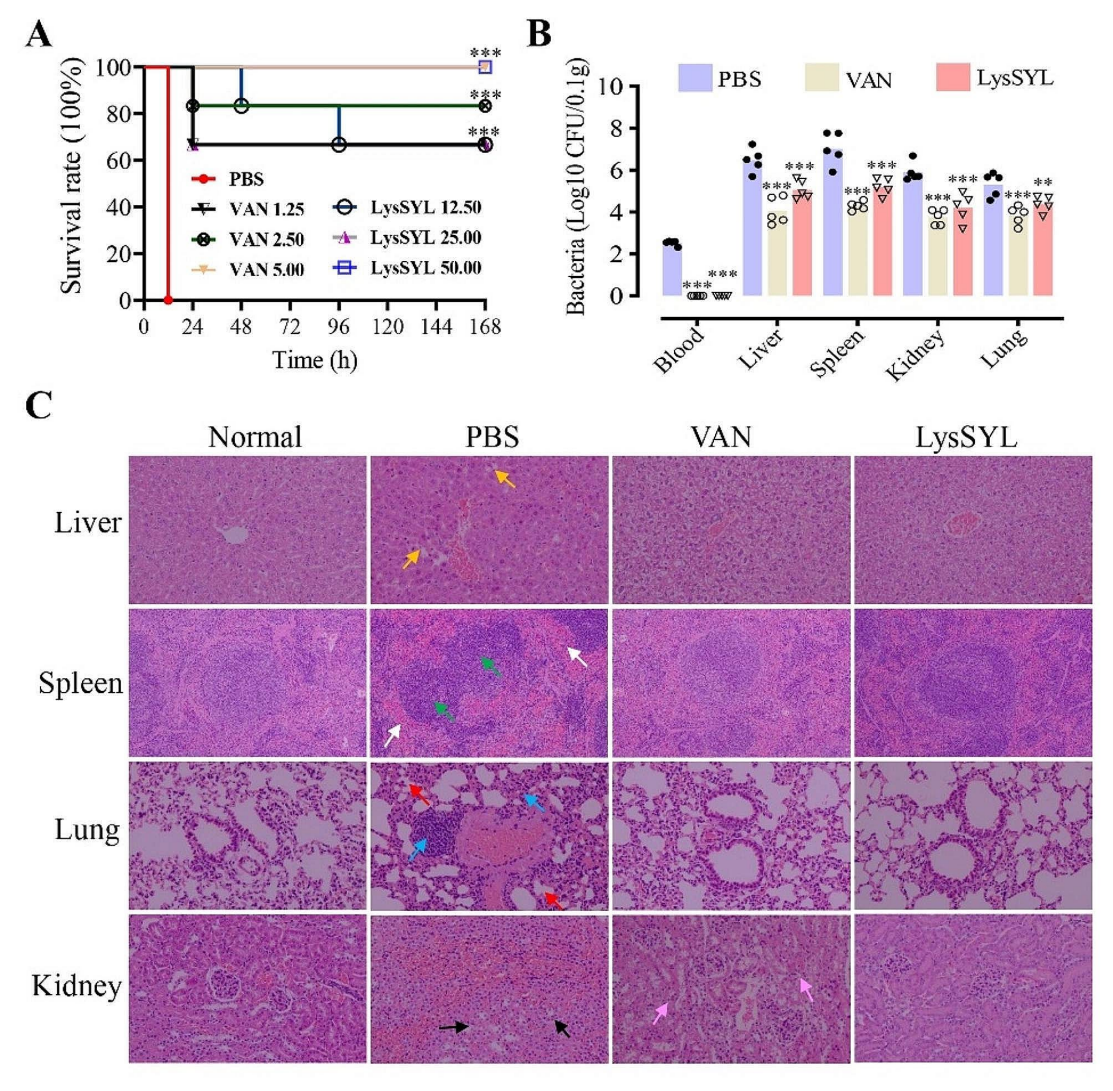
Protection efficacy of LysSYL in a mouse peritonitis model. **A** Survival of mice. Mice (*n* = 6 for each group) were intraperitoneally infected with *S. aureus* USA300 (10^8^ CFU) and treated with LysSYL (12.5, 25, or 50 mg/kg) or VAN (1.25, 2.5, or 5 mg/kg) after 1 h of infection. PBS-treated mice were used as control. Mouse survival was recorded for 7 days. The analyses were measured by simple survival analysis (Kaplan-Meier). *P < 0.05, **P < 0.01, and ***P < 0.001. **B** Bacterial count in the indicated mouse samples. *S. aureus* USA300 (10^8^ CFU) infected mice (*n* = 5) were treated one time with LysSYL (50 mg/kg) or VAN (5 mg/kg), and the blood sample, liver, spleen, lungs, and kidneys were collected after 24 h of treatment and subjected to bacterial counting. PBS-treated mice used as negative control. The analyses were measured by two-way ANOVA. *P < 0.05, **P < 0.01, ***P < 0.001, and ns indicated no significance relative to the PBS control. **C** Protection efficacy of LysSYL on organ injury. Liver, spleen, lungs, and kidneys were harvested from mice sacrificed at 3 d after infection. Unclear hepatic lobule structures, disordered hepatocytes, and hepatic sinusoidal dilation were indicated by yellow arrows. Splenic red pulp congestion and reactive enlarged splenic nodules were indicated by white arrows and green arrows, respectively. Diffuse infiltration of inflammatory cells and shrunken alveolar cavities were shown by blue arrows and red arrows, respectively. Atrophic renal tubules were indicated by black arrows. Edema and degeneration of renal tubular epithelial cells were showed by pink arrows

Next, a mouse peritonitis model was generated (*n* = 6 for each group) by infecting with 1 × 10^8^ CFU of *S. aureus* USA300 and treated with diverse concentrations of LysSYL (12.5, 25, or 50 mg/kg). All mice treated with PBS had died within 12 h post-infection (Fig. 8A). However, treatment with a single dose of LysSYL (12.5 mg/kg) protected 66.7% of the mice 7 days post-infection, and injection of 50 mg/kg of LysSYL rescued 100% of the mice, an effect comparable to that of 5 mg/kg VAN therapy (100% of the mice survived). Bacterial counting revealed that intraperitoneally challenged *S. aureus* (10^8^ CFU) successfully disseminated to most organs, including the liver (6.47 Log10 CFU/0.1 g tissue), spleen (7.02), lungs (5.32), and kidneys (5.90), as well as the bloodstream (2.53) after 12 h of infection (Fig. 8B). Treatment with a single dose of 50 mg/kg LysSYL resulted in a significantly reduction in bacterial loads in the blood (0), liver (5.05), spleen (5.19), lungs (4.37), and kidneys (4.20) of mice infected for 12 h when compared with the PBS control. Injection of 5 mg/kg VAN showed similar bacterial numbers in the tested sample, with the exception of the liver and spleen (Fig. 8B).

Histological analysis revealed significant damage in the liver, spleen, lungs, and kidneys of mice treated with PBS. This damage included unclear hepatic lobule structures, disordered hepatocytes, hepatic sinusoidal dilation, splenic red pulp congestion, reactive enlarged splenic nodules, diffuse infiltration of inflammatory cells, shrunken alveolar cavities, and edema and degeneration of renal tubular epithelial cells with atrophic renal tubules (Fig. 8C). However, LysSYL treatment noticeably alleviated the damage in the spleen, lungs, and kidneys, with no obvious pathological changes when compared with the VAN-treated group.

## Discussion

The AMR crisis has facilitated the exploration of antibiotic alternatives in therapeutic regimens. Numerous alternative treatment strategies are currently under investigation, including quorum sensing inhibition [39], anti-virulence factors [40], antimicrobial oligonucleotides [41], monoclonal antibodies [42], phage therapy [43], and bacteriocins [44]. Among these options, phage therapy using active bacterial viruses or their lytic endolysins appears poised for long-term success; phages are considered inexhaustible, inexpensive, and reformable [41]. However, the screening of phage agents with highly efficient bactericidal activities remains challenging. Most lytic phages are obtained from single samples [16, 27]. In this study, an enrichment procedure was used, and the concentrated sample obtained through tangential flow allowed for the highly efficient isolation of phages with diverse lytic phenotypes (Fig. 1A). Phage SYL, which could form clear and transparent plaques in plates cultured with the MRSA strain XN108, was characterized as a new member of the the *Kayvirus* genus, *Herelleviridae* family, which shows typical characteristics of Twort-like phages such as *Listeria* phage A511 [45].

Some endolysins from *Staphylococcus* phages have been studied, such as ClyS, MV-L, LysK, LysWMY, SAL200, LysH5, and LysGH15 [19, 46]. The endolysin LysSYL consists of two N-terminal CDs (CHAP and Amidase 2) and a C-terminal CBD (SH3b) (Fig. 1E), which represents the typical architecture of phage endolysins [19]. Our data indicate that 50 µg/mL LysSYL can reduce the viable bacteria of XN108 by 6 log10 units (Fig. 3A). By contrast, 250 µg/mL of LysP108, a previously characterized endolysin, only resulted in a reduction of 2 log10 units in viable XN108 numbers [21]. The higher activity of LysSYL may be ascribed to an additional CHAP domain in its structure compared with LysP108. Studies have demonstrated that endolysins containing only the amidase domain often exhibit low lytic activity, and those carrying an extra CHAP domain can increase their lytic activity [47, 48].

Phages generally present a specific host range, whereas endolysins possess a much broader lytic spectrum than their parental phages [49, 50]. Our results support these existing conclusions by showing that LysSYL could reduce the viable bacterial numbers (+++) by over 4 log10 units across all 115 tested strains (100%), including *S. epidermidis*, *S. haemolyticus*, *S. hominis*, *S. capitis*, and most major clones of *S. aureus*. However, phage SYL efficiently lysed only 41.7% of the tested bacteria (+++) (Fig. 3C). One reason for this differing lytic range is the mechanisms used by active phages and endolysins to execute their bactericidal activities. Endolysins, when applied externally, lyse bacterial peptidoglycan from the outside of the cell by applying a method known as “lysis-from-without”. This approach is opposed to the “lysis-from-within” method used by phages, which need suitable receptors on the bacterial surface and hosts to finish their life cycle [51]. Although the endolysins carry identical domains, their lytic spectrums can be variable. LysGH15 is a novel endolysin from staphylococcal phage GH15 and features CHAP, amidase-2, and SH3b domains like LysSYL [46]. However, lytic spectrum determination showed that LysGH15 could only result in 69.8% (37/53) of the tested *S. aureus* isolates by over 4 log10 units of reduction in viable bacteria, indicating a narrow lytic spectrum relative to LysSYL (100%, Fig. 3C). Moreover, LysSYL could rapidly kill *S. aureus* cells (Fig. 4). This rapid bacterial activity is considered a main advantage of phage endolysins [52]. Nelson et al. showed that 107 group A streptococci were reduced to undetectable levels within 10 s after endolysin treatment [53]. Within 30 s, 100 units of endolysin Pal could decrease the viable titer of *S. pneumoniae* by 4.0 log10 units compared with the buffer alone [54]. However, the susceptibility of *S. aureus* to LysSYL may vary by strain, and MRSA XN108 appeared to be more resistant than MRSA USA300 and N315 (Fig. 3A). MRSA XN108 is also a VISA strain [24]. In this case, the thick cell wall associated with VISA may contribute to the slightly increased resistance after LysSYL treatment. The endolysin LysSYL, with its wide lytic spectrum and fast-killing ability, holds a strong promise as a candidate for further applications.

*S. aureus* is popular for its ability to form biofilms, which is a major challenge in eradicating *S. aureus* in clinical settings and often results in the failure of antibiotic treatments [55, 56]. Endolysins are known for their exceptional proficiency in eliminating biofilm-associated infections [57]. Our results reveal that LysSYL exhibits remarkable activity against 24 h- and 72 h-established *S. aureus* biofilms in a concentration-dependent manner (Fig. 5). Removal of 72 h-old biofilms required a longer treatment time than that of the 24 h-old ones (Fig. 5C, D), which is probably due to the presence of a greater amount of extracellular material in the older biofilms. Similar finding has been reported in the case of a chimeric lysin, ClyH [58]. Moreover, LysSYL effectively disrupted mixed-species biofilms formed by *S. aureus* and *A. baumannii* (Fig. 7), which is a common co-infection encountered in clinical practice [13]. However, LysSYL did not exhibit any effect on the disruption of mono-species biofilms generated by *A. baumannii* ATCC 19606 and the CRAB strain AB2 (Fig. 7C, D), which verified the specificity of the endolysin. These data suggest that the eradication of *S. aureus* and *A. baumannii* dual biofilms by LysSYL depends on its ability to target and eliminate *S. aureus*. Polymicrobial biofilms associated with *S. aureus* can be effectively dismantled by targeting the main pathogen to induce biofilm collapse and clearance [32], suggesting that anti-*S. aureus* activity alone can disrupt polymicrobial biofilms. Furthermore, the eradication activity of LysSYL against mono-species *S. aureus* biofilms was more potent than that for polymicrobial biofilms. The OD595 values were 1.79 and 2.46 in polymicrobial biofilms after treatment with 32 µg/mL of LysSYL (Fig. 7A, C), respectively, which were much higher than in mono-species *S. aureus* biofilms after treatment with LysSYL (0.15) (Fig. 5C). The presence of cells of a nonsensitive species in the biofilms may hinder the ability of LysSYL to lyse the target cells, and several mechanisms might be involved, such as interspecies signaling, spatial distribution of physiologically different bacteria, and interference from the matrix [59]. With the biofilm disrupted by LysSYL, conventional antimicrobials and host innate immunity can subsequently kill the remaining planktonic bacteria [12]. Using a mouse peritonitis model, we further demonstrated that a single intraperitoneal injection of LysSYL (50 mg/kg) can rescue all the mice challenged with a lethal dose of MRSA USA300 (10^8^ CFU, Fig. 8). This finding suggests that LysSYL is a promising candidate for the development of efficient therapeutic regimens to control *S. aureu*s infections. However, the overall safety and treatment value of endolysin LysSYL need further extensive investigation.

## Conclusions

In conclusion, the implementation of the enrichment procedure allowed for the isolation of more desirable phages. Subsequently, an endolysin known as LysSYL, which originates from the screened phage SYL that belongs to the *Kayvirus* genus, *Herelleviridae* family, was characterized and expressed. The antibacterial effects of LysSYL were confirmed in vitro and in vivo. LysSYL exhibited broad-spectrum bactericidal activity against bacteria within the *Staphylococcus* genus and showcased rapid killing capabilities. Its remarkable ability to combat biofilms suggests a promising application in the management of *S. aureus* infections associated with biofilm formation.

### Electronic supplementary material

Below is the link to the electronic supplementary material.

Additional file 1: **Movie. S1** Dynamic observations of the bactericidal process treated with LysSYL using CLSM.

Additional file 2: **Movie. S2.** Dynamic observations of the bactericidal process treated with PBS using CLSM.

Additional file 3: **Fig. S1.** Phylogenetic analysis of phage SYL and its closely related phages based on the whole genome sequences. **Fig.****S2****.** Phylogenetic analysis of LysSYL and previously characterized endolysins based on protein sequences. **Fig.****S3****.** Identification of the recombinant pET21a-LysSYL expression plasmid. **Fig. S4.** SDS-PAGE analysis of the optimized conditions for LysSYL expression in *E. coli* BL21/pET21a-LysSYL bacteria. **Fig. S5.** Evaluation of bactericidal activity of endolysin LysSYL against *S. aureus* with zone inhibition assay. **Fig. S6.** Evaluation of lytic activity of phage SYL against Gram-negative bacteria with zone inhibition assay. **Fig. S7.** Bactericidal activity of endolysin LysSYL against *S. aureus*. **Fig. S8.** Disruption of mixed-species biofilms associated with *S. aureus*. **Fig. S9.** Safety evaluation of endolysin LysSYL in vivo. **Table****S1****. **Strains used in this study. **Table****S2****.** The MIC values of antimicrobial agents against bacteria.

## Data Availability

No datasets were generated or analysed during the current study.

## Abbreviations

MRSA: Methicillin resistant *S. aureus*
CLSM: Confocal laser scanning microscopy
SEM: Scan electron microscopy
AMR: Antimicrobial resistance
MDR: Multidrug-resistant
VAN: Vancomycin
VISA: Vancomycin-intermediate *S. aureus*
hVISA: Heterogeneous vancomycin-intermediate *S. aureus*
TEM: Transmission electron microscopy
OsO_4_: Osmium tetroxide
IPTG: Isopropyl-β-d-thiogalactoside
SDS-PAGE: Sodium dodecyl sulfate polyacrylamide gel electrophoresis
LPS: Lipopolysaccharide
CFU: Colony forming unit
MSSA: Methicillin-sensitive *S. aureus*
EDTA: Ethylene diamine tetraacetic acid
PBS: Phosphate buffer saline
MIC: Minimal inhibitory concentration
MEM: Meropenem
PFU: Plaque forming unit
PI: Propidium iodide
CEF: Cefuroxime
H&E: Hematoxylin and eosin
MLD: Minimal lethal dose
CDSs: Coding sequences
CDs: Catalytic domains
CHAP: Cysteine- and histidine-dependent aminopeptidase/ hydrolase
Amidase: 2 N-acetylmuramoyl-L-alanine amidase
SH3b: Src homology 3 domain
CBD: Cell wall recognition and binding
