# Supplementary material for: LysSYL: a broad-spectrum phage endolysin targeting Staphylococcus species and eradicating S. aureus biofilms
Source: Microb Cell Fact. 2024 Mar 25;23:89. doi: 10.1186/s12934-024-02359-4 (PMC10962180; doi:10.1186/s12934-024-02359-4)
Supplement: Supplementary file 3 — Additional file 3: Fig. S1. Phylogenetic analysis of phage SYL and its closely related phages based on the whole genome sequences. Fig.S2. Phylogenetic analysis of LysSYL and previously characterized endolysins based on protein sequences. Fig.S3. Identification of the recombinant pET21a-LysSYL expression plasmid. Fig. S4. SDS-PAGE analysis of the optimized conditions for LysSYL expression in E. coli BL21/pET21a-LysSYL bacteria. Fig. S5. Evaluation of bactericidal activity of endolysin LysSYL against S. aureus with zone inhibition assay. Fig. S6. Evaluation of lytic activity of phage SYL against Gram-negative bacteria with zone inhibition assay. Fig. S7. Bactericidal activity of endolysin LysSYL against S. aureus. Fig. S8. Disruption of mixed-species biofilms associated with S. aureus. Fig. S9. Safety evaluation of endolysin LysSYL in vivo. TableS1. Strains used in this study. TableS2. The MIC values of antimicrobial agents against bacteria. [file 12934_2024_2359_MOESM3_ESM.docx]

**Supplementary Materials**

**
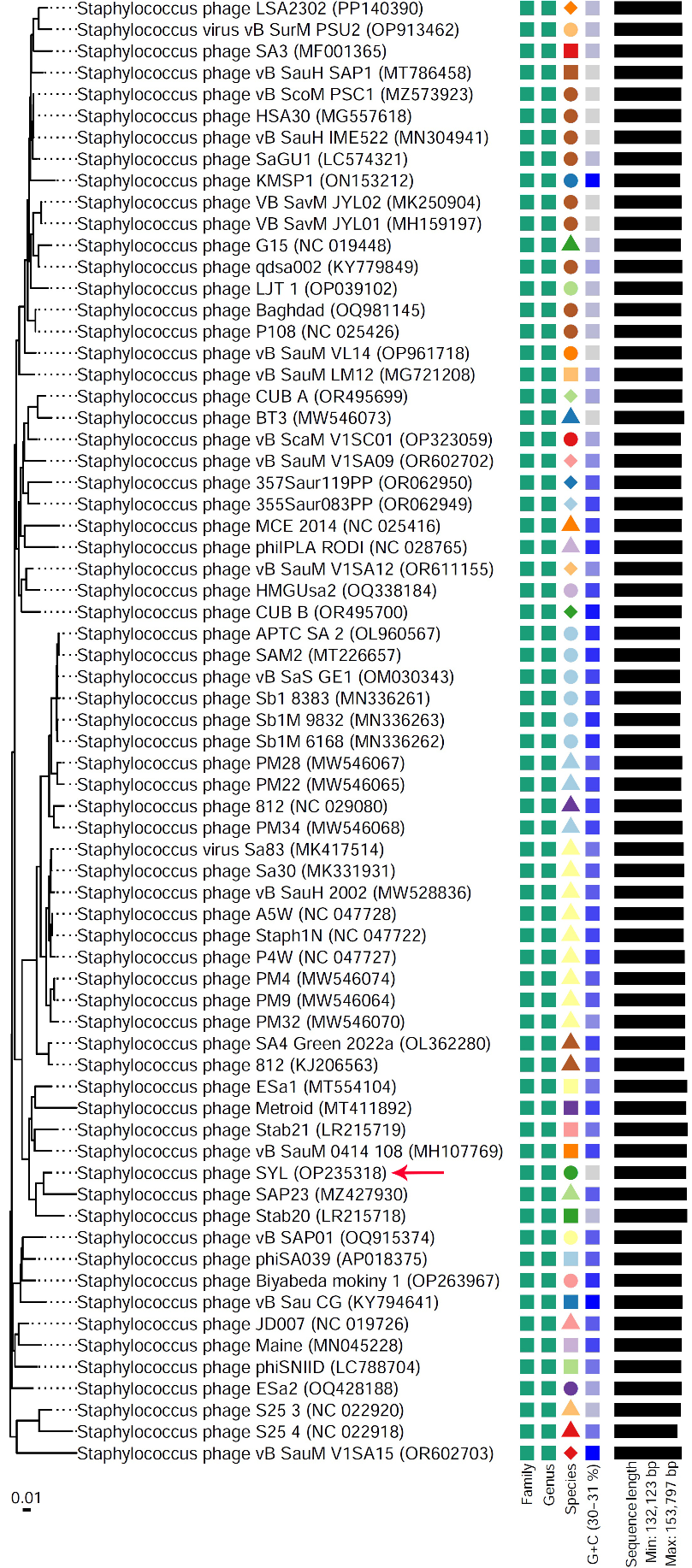
**

**Fig. S1.** Phylogenetic analysis of phage SYL and its closely related phages based on the whole genome sequences. The scale length of relative evolution distance is 0.01. The position of phage SYL is indicated by a red arrow.


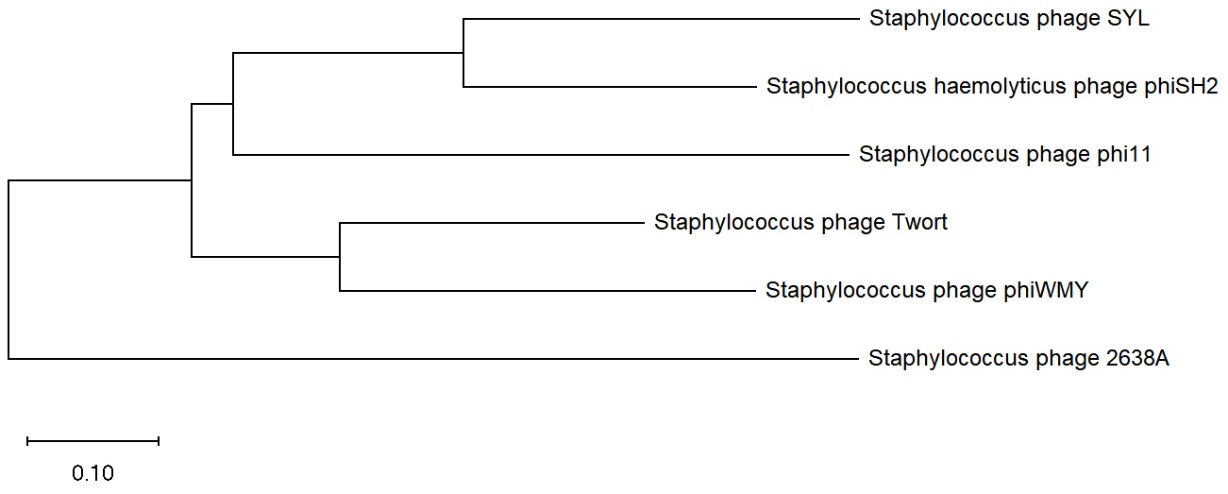


**Fig. S2.** Phylogenetic analysis of LysSYL and previously characterized endolysins based on the protein sequences. The protein sequences of endolysins from phages Twort, phi11, 2638A, phiSH2, and phiWMY were downloaded from NCBI. The multiple sequence alignments of endolysins were conducted using ClustalW with default parameters. The scale length of relative evolution distance is 0.10.


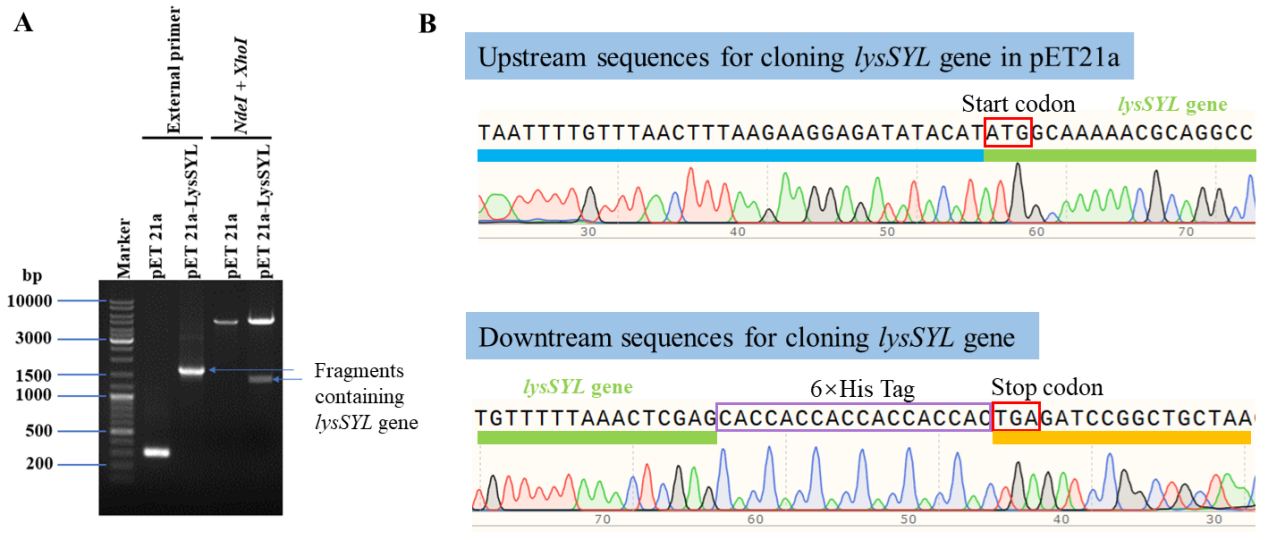


**Fig. S3.** Identification of the recombinant pET21a-LysSYL expression plasmid**.** (**A**) Characterization of pET21a-LysSYL by PCR and restriction enzyme analysis. Fragments were amplified with external primers using pET21a-LysSYL or pET21a empty vector as templates. The pET21a-LysSYLwas digested by NdeI and XhoI. The resulting bands indicated the fragments containing *lysSYL* gene (blue arrows). (**B**) DNA sequencing of plasmid pET21a-LysSYL. The full-length of target gene is 1494 bp. The sequence of *lysSYL* gene was indicated in green, and the upstream and downstream DNA sequences locating on the pET21a vector were shown in blue and yellow, respectively. The 6×His Tag, start codon, and stop codon were indicated.


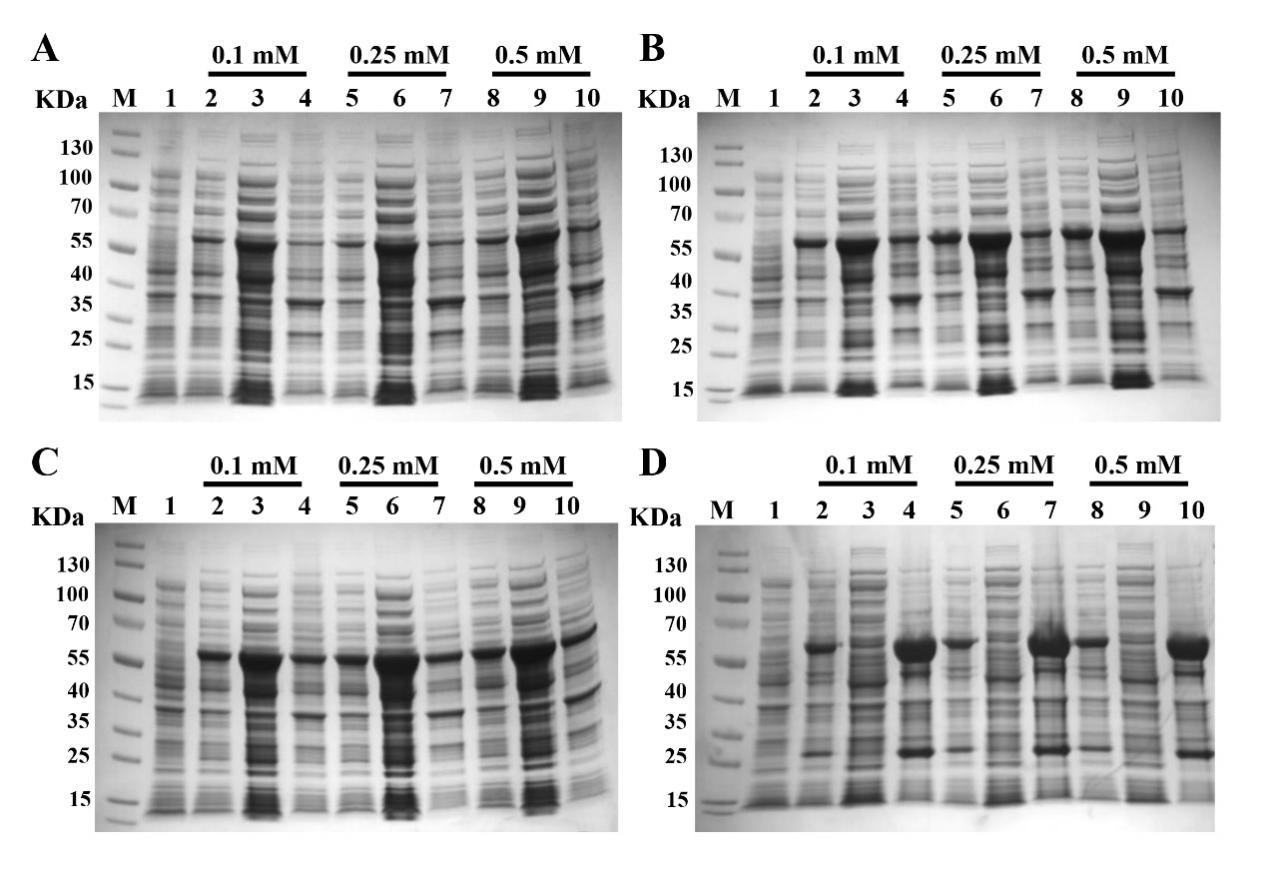


**Fig. S4.** SDS-PAGE analysis of the optimized conditions for LysSYL expression in *E. coli* BL21/pET21a-LysSYL bacteria. *E. coli* BL21/pET21a-LysSYL bacteria were cultured at (**A**) 16 °C for 16 h, (**B**) 23 °C for 16 h, (**C**) 30 °C for 5 h, and (**D**) 37 °C for 5 h after induction with 0.1, 0.25, and 0.5 mM IPTG as indicated. M, protein molecular marker; Lane 1, uninduced *E. coli* BL21/pET21a-LysSYL bacteria (negative control); Lanes 2–4, 5–7, and 8–10 represented bacteria induced with 0.1, 0.25, and 0.5 mM IPTG, respectively. Lanes 2, 5, and 8, the supernatant after sonication of the induced bacteria; Lanes 3, 6, and 9, protein pellet lysate, and Lanes 4, 7, and 10, protein pellets.


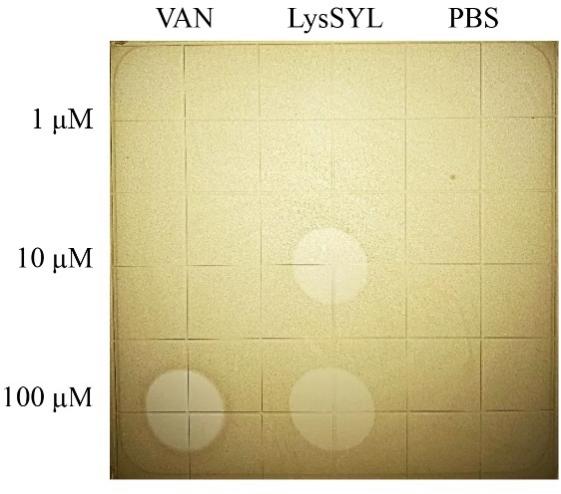


**Fig. S5.** Evaluation of bactericidal activity of endolysin LysSYL against *S. aureus* with zone inhibition assay. *S. aureus* XN108 was cultured and inoculated to a BHI agar plate. About 20 µL of LysSYL (1, 10, and 100 µM, respectively) were dropped. VAN served as positive control, and PBS represented negative control. The inhibition zones were observed after culture the plate at 37 °C for 16 h.


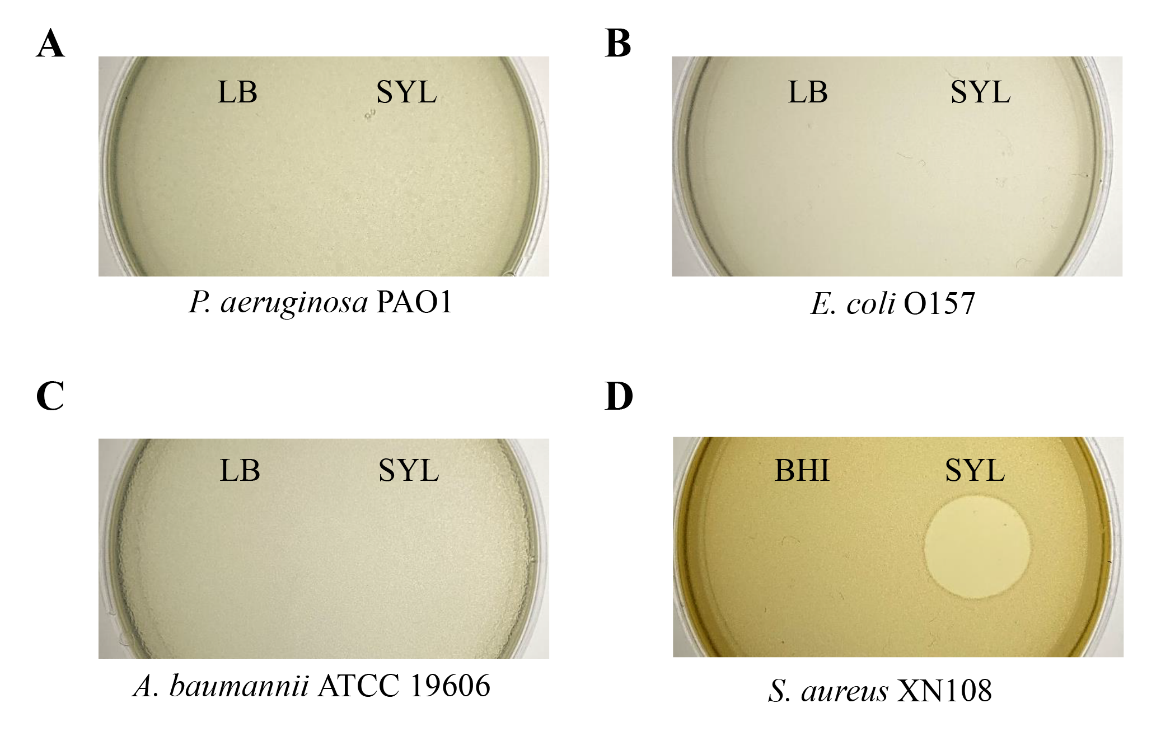


**Fig. S6.** Evaluation of lytic activity of phage SYL against Gram-negative bacteria with zone inhibition assay. Bactericidal activities of phage SYL against (**A**) *P. aeruginosa*, (**B**) *E. coli*, (**C**) *A. baumannii*, and (**D**) *S. aureus* XN108 (positive control). The inhibition zones were observed after culture of the plates at 37 °C for 16 h.

**
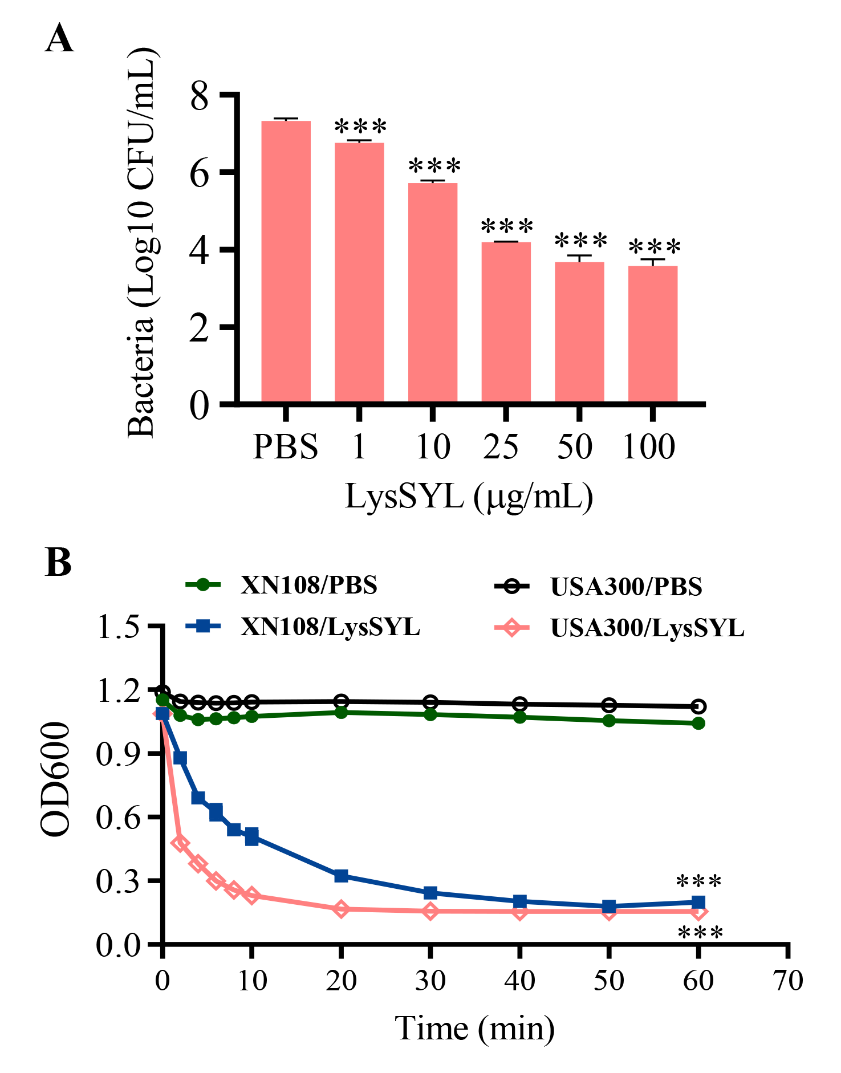
**

**Fig. S7.** Bactericidal activity of endolysin LysSYL against *S. aureus*. **A** CFU reduction assay for bactericidal activity of LysSYL against *S. aureus* XN108. Different concentrations of LysSYL were used, and PBS served as control. Significance was calculated by one-way ANOVA between LysSYL treatment and PBS control. ****P*< 0.0001. **B** Lytic activity detection of endolysin LysSYL against *S. aureus* XN108 and *S. aureus* USA300. Bacterial cultures were treated with 50 μg/mL LysSYL for 1 h. OD600 values were measured every 2 min. PBS was used as a control. Each test was assayed in triplicate. Data were expressed as mean ± SD. The statistical analysis was measured by two-way ANOVA. ****P*<0.001.


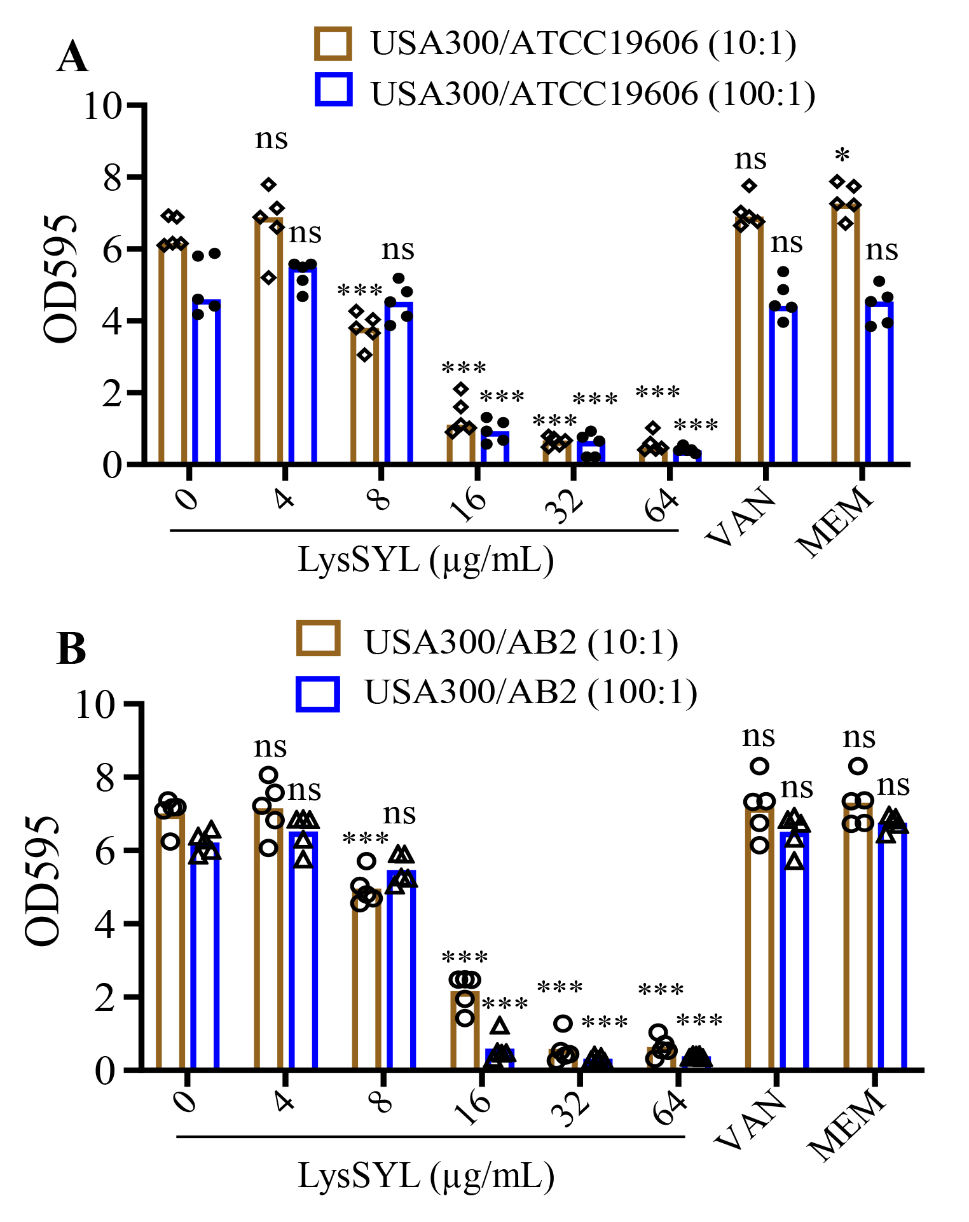


**Fig. S8.** Disruption of mixed-species biofilms associated with *S. aureus*. Disruption of biofilms established with (**A**) *S. aureus* USA300 and *A. baumannii* ATCC 19606 in ratios of 10:1 and 100:1, respectively, and (**B**) *S. aureus* USA300 and *A. baumannii* AB2 in ratios of 10:1 and 100:1, respectively. The biofilms were treated with 200 μL of LysSYL (4–64 μg/mL), VAN (64 μg/mL), and MEM (128 μg/mL for *A. baumannii* ATCC 19606, and 8.192 mg/mL for AB2) for 1 h. The experiment was conducted three times. Data were expressed as mean ± SD. The analyses were measured by two-way ANOVA. **P*<0.05, ****P*<0.001, and ns represents no significance.


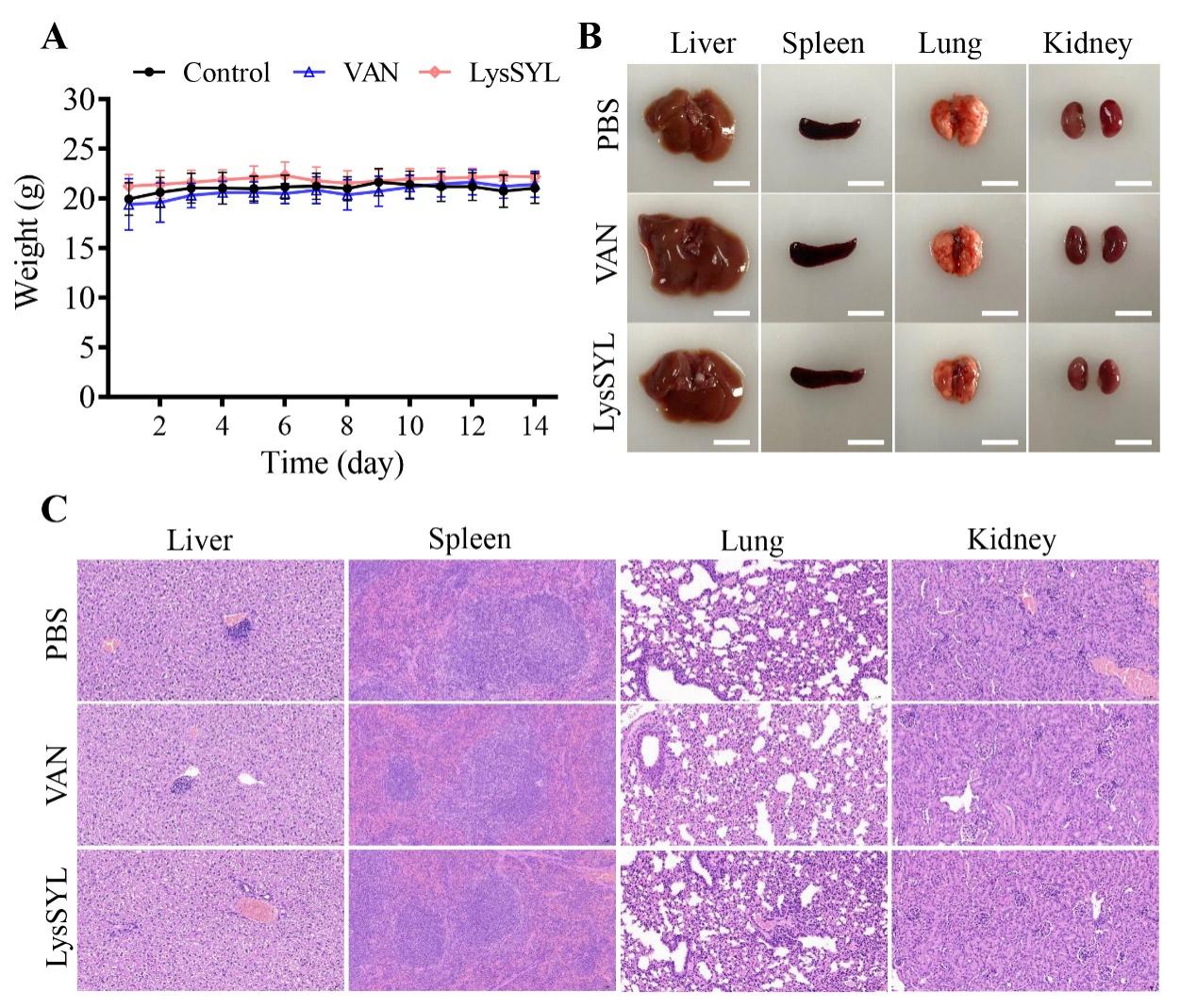


**Fig. S9.** Safety evaluation of endolysin LysSYL *in vivo*. **A** Body weight variation after challenged with LysSYL. BALB/c mice (*n*=5 per group) were intraperitoneally injected with 50 mg/kg LysSYL, or 5 mg/kg VAN. PBS served as negative control. Body weights of the challenged mice were monitored up to 14 days. Data were expressed as mean ± SD. **B** Photographs of organs harvested at 14 d after exposure to 50 mg/kg LysSYL or 5 mg/kg VAN. PBS served as control. Scale bars, 1 cm. **C** Histological analysis of mouse organs harvested at 14 d after exposure to 50 mg/kg LysSYL or 5 mg/kg VAN.

**Table S1 Strains used in this study.**

| **Stains** | **MLST type** | **phenotype** | **Description** | **Source** **or reference** |
| --- | --- | --- | --- | --- |
| ***Staphylococcus aureus*** | | | | |
| TJ008 | ST59 | MRSA | A clinical strain isolated in Tianjin, susceptible to tigecycline, vancomycin, and linezolid | [1] |
| TJ024 | ST59 | MRSA | A clinical strain isolated in Tianjin, susceptible to tigecycline, vancomycin, and linezolid | [1] |
| TJ026 | ST59 | MRSA | A clinical strain isolated in Tianjin, susceptible to tigecycline, vancomycin, and linezolid | [1] |
| TJ034 | ST59 | MRSA | A clinical strain isolated in Tianjin, susceptible to tigecycline, vancomycin, and linezolid | [1] |
| TJ044 | ST59 | MRSA | A clinical strain isolated in Tianjin, susceptible to tigecycline, vancomycin, and linezolid | [1] |
| TJ035 | ST59 | MSSA | A clinical strain isolated in Tianjin, susceptible to tigecycline, vancomycin, and linezolid | [1] |
| TJ036 | ST59 | MSSA | A clinical strain isolated in Tianjin, susceptible to tigecycline, vancomycin, and linezolid | [1] |
| TJ109 | ST59 | MSSA | A clinical strain isolated in Tianjin, susceptible to tigecycline, vancomycin, and linezolid | [1] |
| TJ110 | ST59 | MSSA | A clinical strain isolated in Tianjin, susceptible to tigecycline, vancomycin, and linezolid | [1] |
| TJ111 | ST59 | MSSA | A clinical strain isolated in Tianjin, susceptible to tigecycline, vancomycin, and linezolid | [1] |
| TJ093 | ST398 | MRSA | A clinical strain isolated in Tianjin, susceptible to tigecycline, vancomycin, and linezolid | [1] |
| TJ136 | ST398 | MRSA | A clinical strain isolated in Tianjin, susceptible to tigecycline, vancomycin, and linezolid | [1] |
| TJ161 | ST398 | MRSA | A clinical strain isolated in Tianjin, susceptible to tigecycline, vancomycin, and linezolid | [1] |
| TJ163 | ST398 | MRSA | A clinical strain isolated in Tianjin, susceptible to tigecycline, vancomycin, and linezolid | [1] |
| TJ174 | ST398 | MRSA | A clinical strain isolated in Tianjin, susceptible to tigecycline, vancomycin, and linezolid | [1] |
| TJ003 | ST398 | MSSA | A clinical strain isolated in Tianjin, susceptible to tigecycline, vancomycin, and linezolid | [1] |
| TJ013 | ST398 | MSSA | A clinical strain isolated in Tianjin, susceptible to tigecycline, vancomycin, and linezolid | [1] |
| TJ020 | ST398 | MSSA | A clinical strain isolated in Tianjin, susceptible to tigecycline, vancomycin, and linezolid | [1] |
| TJ025 | ST398 | MSSA | A clinical strain isolated in Tianjin, susceptible to tigecycline, vancomycin, and linezolid | [1] |
| TJ033 | ST398 | MSSA | A clinical strain isolated in Tianjin, susceptible to tigecycline, vancomycin, and linezolid | [1] |

**Table S1** *(Continued)*

| **Stain** | **MLST type** | **Resistance** | **Description** | **Source** **or reference** |
| --- | --- | --- | --- | --- |
| TJ005 | ST188 | MRSA | A clinical strain isolated in Tianjin, susceptible to tigecycline, vancomycin, and linezolid | [1] |
| TJ006 | ST188 | MSSA | A clinical strain isolated in Tianjin, susceptible to tigecycline, vancomycin, and linezolid | [1] |
| TJ022 | ST188 | MSSA | A clinical strain isolated in Tianjin, susceptible to tigecycline, vancomycin, and linezolid | [1] |
| TJ037 | ST188 | MSSA | A clinical strain isolated in Tianjin, susceptible to tigecycline, vancomycin, and linezolid | [1] |
| TJ040 | ST188 | MSSA | A clinical strain isolated in Tianjin, susceptible to tigecycline, vancomycin, and linezolid | [1] |
| TJ049 | ST188 | MSSA | A clinical strain isolated in Tianjin, susceptible to tigecycline, vancomycin, and linezolid | [1] |
| TJ056 | ST188 | MSSA | A clinical strain isolated in Tianjin, susceptible to tigecycline, vancomycin, and linezolid | [1] |
| TJ118 | ST188 | MSSA | A clinical strain isolated in Tianjin, susceptible to tigecycline, vancomycin, and linezolid | [1] |
| TJ157 | ST188 | MSSA | A clinical strain isolated in Tianjin, susceptible to tigecycline, vancomycin, and linezolid | [1] |
| TJ171 | ST188 | MSSA | A clinical strain isolated in Tianjin, susceptible to tigecycline, vancomycin, and linezolid | [1] |
| TJ095 | ST1 | MRSA | A clinical strain isolated in Tianjin, susceptible to tigecycline, vancomycin, and linezolid | [1] |
| TJ187 | ST1 | MRSA | A clinical strain isolated in Tianjin, susceptible to tigecycline, vancomycin, and linezolid | [1] |
| TJ002 | ST1 | MSSA | A clinical strain isolated in Tianjin, susceptible to tigecycline, vancomycin, and linezolid | [1] |
| TJ004 | ST1 | MSSA | A clinical strain isolated in Tianjin, susceptible to tigecycline, vancomycin, and linezolid | [1] |
| TJ010 | ST1 | MSSA | A clinical strain isolated in Tianjin, susceptible to tigecycline, vancomycin, and linezolid | [1] |
| TJ060 | ST1 | MSSA | A clinical strain isolated in Tianjin, susceptible to tigecycline, vancomycin, and linezolid | [1] |
| TJ086 | ST1 | MSSA | A clinical strain isolated in Tianjin, susceptible to tigecycline, vancomycin, and linezolid | [1] |
| TJ094 | ST1 | MSSA | A clinical strain isolated in Tianjin, susceptible to tigecycline, vancomycin, and linezolid | [1] |
| TJ114 | ST1 | MSSA | A clinical strain isolated in Tianjin, susceptible to tigecycline, vancomycin, and linezolid | [1] |
| TJ117 | ST1 | MSSA | A clinical strain isolated in Tianjin, susceptible to tigecycline, vancomycin, and linezolid | [1] |

**Table S1** *(Continued)*

| **Stain** | **MLST type** | **Resistance** | **Description** | **Source** **or reference** |
| --- | --- | --- | --- | --- |
| TJ102 | ST5 | MRSA | A clinical strain isolated in Tianjin, susceptible to tigecycline, vancomycin, and linezolid | [1] |
| SH46 | ST5 | MRSA | A clinical *S. aureus* strain isolated in Shanghai from 2009 to 2012 | [2] |
| N315 | ST5 | MRSA | GenBank accession. no. NC_002745.2, partly resistant to β–lactam antibiotics and susceptible to vancomycin, | [3] |
| TJ039 | ST5 | MSSA | A clinical strain isolated in Tianjin, susceptible to tigecycline, vancomycin, and linezolid | [1] |
| TJ099 | ST5 | MSSA | A clinical strain isolated in Tianjin, susceptible to tigecycline, vancomycin, and linezolid | [1] |
| TJ121 | ST5 | MSSA | A clinical strain isolated in Tianjin, susceptible to tigecycline, vancomycin, and linezolid | [1] |
| TJ132 | ST5 | MSSA | A clinical strain isolated in Tianjin, susceptible to tigecycline, vancomycin, and linezolid | [1] |
| TJ144 | ST5 | MSSA | A clinical strain isolated in Tianjin, susceptible to tigecycline, vancomycin, and linezolid | [1] |
| TJ147 | ST5 | MSSA | A clinical strain isolated in Tianjin, susceptible to tigecycline, vancomycin, and linezolid | [1] |
| TJ149 | ST5 | MSSA | A clinical strain isolated in Tianjin, susceptible to tigecycline, vancomycin, and linezolid | [1] |
| TJ167 | ST5 | MSSA | A clinical strain isolated in Tianjin, susceptible to tigecycline, vancomycin, and linezolid | [1] |
| TJ014 | ST5527 | MRSA | A clinical strain isolated in Tianjin, susceptible to tigecycline, vancomycin, and linezolid | [1] |
| TJ017 | ST5527 | MRSA | A clinical strain isolated in Tianjin, susceptible to tigecycline, vancomycin, and linezolid | [1] |
| TJ030 | ST5527 | MRSA | A clinical strain isolated in Tianjin, susceptible to tigecycline, vancomycin, and linezolid | [1] |
| TJ041 | ST5527 | MRSA | A clinical strain isolated in Tianjin, susceptible to tigecycline, vancomycin, and linezolid | [1] |
| TJ042 | ST5527 | MRSA | A clinical strain isolated in Tianjin, susceptible to tigecycline, vancomycin, and linezolid | [1] |
| TJ043 | ST5527 | MRSA | A clinical strain isolated in Tianjin, susceptible to tigecycline, vancomycin, and linezolid | [1] |
| TJ050 | ST5527 | MRSA | A clinical strain isolated in Tianjin, susceptible to tigecycline, vancomycin, and linezolid | [1] |
| TJ053 | ST5527 | MRSA | A clinical strain isolated in Tianjin, susceptible to tigecycline, vancomycin, and linezolid | [1] |
| TJ080 | ST5527 | MRSA | A clinical strain isolated in Tianjin, susceptible to tigecycline, vancomycin, and linezolid | [1] |

**Table S1** *(Continued)*

| **Stain** | **MLST type** | **Resistance** | **Description** | **Source** **or reference** |
| --- | --- | --- | --- | --- |
| TJ090 | ST5527 | MRSA | A clinical strain isolated in Tianjin, susceptible to tigecycline, vancomycin, and linezolid | [1] |
| TJ027 | ST22 | MSSA | A clinical strain isolated in Tianjin, susceptible to tigecycline, vancomycin, and linezolid | [1] |
| TJ038 | ST22 | MSSA | A clinical strain isolated in Tianjin, susceptible to tigecycline, vancomycin, and linezolid | [1] |
| TJ048 | ST22 | MSSA | A clinical strain isolated in Tianjin, susceptible to tigecycline, vancomycin, and linezolid | [1] |
| TJ059 | ST22 | MSSA | A clinical strain isolated in Tianjin, susceptible to tigecycline, vancomycin, and linezolid | [1] |
| TJ062 | ST22 | MSSA | A clinical strain isolated in Tianjin, susceptible to tigecycline, vancomycin, and linezolid | [1] |
| TJ071 | ST22 | MSSA | A clinical strain isolated in Tianjin, susceptible to tigecycline, vancomycin, and linezolid | [1] |
| TJ076 | ST22 | MSSA | A clinical strain isolated in Tianjin, susceptible to tigecycline, vancomycin, and linezolid | [1] |
| TJ078 | ST22 | MSSA | A clinical strain isolated in Tianjin, susceptible to tigecycline, vancomycin, and linezolid | [1] |
| TJ083 | ST22 | MSSA | A clinical strain isolated in Tianjin, susceptible to tigecycline, vancomycin, and linezolid | [1] |
| TJ085 | ST22 | MSSA | A clinical strain isolated in Tianjin, susceptible to tigecycline, vancomycin, and linezolid | [1] |
| TJ001 | ST25 | MRSA | A clinical strain isolated in Tianjin, susceptible to tigecycline, vancomycin, and linezolid | [1] |
| TJ097 | ST25 | MRSA | A clinical strain isolated in Tianjin, susceptible to tigecycline, vancomycin, and linezolid | [1] |
| TJ106 | ST25 | MRSA | A clinical strain isolated in Tianjin, susceptible to tigecycline, vancomycin, and linezolid | [1] |
| TJ209 | ST25 | MRSA | A clinical strain isolated in Tianjin, susceptible to tigecycline, vancomycin, and linezolid | [1] |
| TJ011 | ST25 | MSSA | A clinical strain isolated in Tianjin, susceptible to tigecycline, vancomycin, and linezolid | [1] |
| TJ021 | ST25 | MSSA | A clinical strain isolated in Tianjin, susceptible to tigecycline, vancomycin, and linezolid | [1] |
| TJ023 | ST25 | MSSA | A clinical strain isolated in Tianjin, susceptible to tigecycline, vancomycin, and linezolid | [1] |
| TJ028 | ST25 | MSSA | A clinical strain isolated in Tianjin, susceptible to tigecycline, vancomycin, and linezolid | [1] |
| TJ031 | ST25 | MSSA | A clinical strain isolated in Tianjin, susceptible to tigecycline, vancomycin, and linezolid | [1] |

**Table S1** *(Continued)*

| **Stain** | **MLST type** | **Resistance** | **Description** | **Source** **or reference** |
| --- | --- | --- | --- | --- |
| TJ051 | ST25 | MSSA | A clinical strain isolated in Tianjin, susceptible to tigecycline, vancomycin, and linezolid | [1] |
| GZ11 | ST239 | MRSA | A clinical strain isolated in Guangzhou | [2] |
| GZ21 | ST239 | MRSA | A clinical strain isolated in Guangzhou | [2] |
| GZ23 | ST239 | MRSA | A clinical strain isolated in Guangzhou | [2] |
| GZ26 | ST239 | MRSA | A clinical strain isolated in Guangzhou | [2] |
| GZ28 | ST239 | MRSA | A clinical strain isolated in Guangzhou | [2] |
| GZ29 | ST239 | MRSA | A clinical strain isolated in Guangzhou | [2] |
| GZ31 | ST239 | MRSA | A clinical strain isolated in Guangzhou | [2] |
| GZ34 | ST239 | MRSA | A clinical strain isolated in Guangzhou | [2] |
| GZ38 | ST239 | MRSA | A clinical strain isolated in Guangzhou | [2] |
| GZ56 | ST239 | MRSA | A clinical strain isolated in Guangzhou | [2] |
| GZ11 | ST239 | MRSA | A clinical strain isolated in Guangzhou | [2] |
| GZ21 | ST239 | MRSA | A clinical strain isolated in Guangzhou | [2] |
| GZ23 | ST239 | MRSA | A clinical strain isolated in Guangzhou | [2] |
| GZ26 | ST239 | MRSA | A clinical strain isolated in Guangzhou | [2] |
| GZ28 | ST239 | MRSA | A clinical strain isolated in Guangzhou | [2] |
| GZ29 | ST239 | MRSA | A clinical strain isolated in Guangzhou | [2] |
| GZ31 | ST239 | MRSA | A clinical strain isolated in Guangzhou | [2] |
| GZ34 | ST239 | MRSA | A clinical strain isolated in Guangzhou | [2] |
| GZ38 | ST239 | MRSA | A clinical strain isolated in Guangzhou | [2] |
| GZ56 | ST239 | MRSA | A clinical strain isolated in Guangzhou | [2] |
| XN108 | ST239 | MRSA | A clinical vancomycin-intermediate *S. aureus* strain (VISA) carrying WalK(S221P), GraS(T136I), and RpoB(H481N) mutations in its genome. GenBank accession no. CP007447.1 | [4] |
| TJ103 | ST6 | MRSA | A clinical strain isolated in Tianjin, susceptible to tigecycline, vancomycin, and linezolid | [1] |
| TJ058 | ST6 | MSSA | A clinical strain isolated in Tianjin, susceptible to tigecycline, vancomycin, and linezolid | [1] |

**Table S1** *(Continued)*

| **Stain** | **MLST type** | | | **Resistance** | **Description** | **Source** **or reference** |
| --- | --- | --- | --- | --- | --- | --- |
| TJ063 | ST8 | | | MSSA | A clinical strain isolated in Tianjin, susceptible to tigecycline, vancomycin, and linezolid | [1] |
| TJ068 | ST8 | | | MSSA | A clinical strain isolated in Tianjin, susceptible to tigecycline, vancomycin, and linezolid | [1] |
| TJ120 | ST15 | | | MRSA | A clinical strain isolated in Tianjin, susceptible to tigecycline, vancomycin, and linezolid | [1] |
| TJ131 | ST15 | | | MSSA | A clinical strain isolated in Tianjin, susceptible to tigecycline, vancomycin, and linezolid | [1] |
| TJ012 | ST72 | | | MRSA | A clinical strain isolated in Tianjin, susceptible to tigecycline, vancomycin, and linezolid | [1] |
| TJ061 | ST72 | | | MSSA | A clinical strain isolated in Tianjin, susceptible to tigecycline, vancomycin, and linezolid | [1] |
| TJ178 | ST338 | | | MRSA | A clinical strain isolated in Tianjin, susceptible to tigecycline, vancomycin, and linezolid | [1] |
| TJ119 | ST338 | | | MSSA | A clinical strain isolated in Tianjin, susceptible to tigecycline, vancomycin, and linezolid | [1] |
| Newman | | ST254 | | MSSA | NCTC 8178, ST8/*agr*-I, highly virulent, extensively used in *S. aureus* animal models | [5] |
| USA300 | | ST8 | | MRSA | ATCC-BAA-1556 (FPR3757), *agr*-IV, highly virulent, a multidrug resistant strain extensively used in *S. aureus* animal models | [6] |
| ATCC 25923 | | ST243 | | MSSA | A clinical isolate used as a standard laboratory testing control strain. GenBank accession no. CP009361.1 | [7] |
| ***Staphylococcus haemolyticus*** | | | | | | |
| RXN117 | | | - | MRCNS | A clinical strain isolated in Chongqing | This study |
| RXN128 | | | - | MRCNS | A clinical strain isolated in Chongqing | This study |
| RXN141 | | | - | MRCNS | A clinical strain isolated in Chongqing | This study |
| RXN154 | | | - | MSCNS | A clinical strain isolated in Chongqing | This study |
| RXN157 | | | - | MRCNS | A clinical strain isolated in Chongqing | This study |
| ***Staphylococcus epidermidis*** | | | | | | |
| BXN1 | - | | | MRCNS | A clinical strain isolated in Chongqing | This study |
| BXN2 | - | | | MRCNS | A clinical strain isolated in Chongqing | This study |
| BXN3 | - | | | MRCNS | A clinical strain isolated in Chongqing | This study |
| BZP1 | - | | | - | A clinical stain isolated from patient scar tissue | This study |

**Table S1** *(Continued)*

| **Stain** | **MLST type** | **Resistance** | **Description** | **Source** **or reference** |
| --- | --- | --- | --- | --- |
| BZP2 | - | - | A clinical *S. epidermidis* stain isolated from patient scar tissue | This study |
| ***Staphylococcus*** ***hominis*** | | | | |
| HXN1 | - | MRCNS | A clinical strain isolated in Chongqing | This study |
| HXN2 | - | MRCNS | A clinical strain isolated in Chongqing | This study |
| HXN3 | - | MRCNS | A clinical strain isolated in Chongqing | This study |
| ***Staphylococcus capitis*** | | | | |
| TXN1 | - | MRCNS | A clinical strain isolated in Chongqing | This study |
| TXN2 | - | MRCNS | A clinical strain isolated in Chongqing | This study |
| ***Pseudomonas aeruginosa*** | | | | |
| PAO1 | ST549 | - | Wild-type *P. aeruginosa* strain PAO1 | [8] |
| PA1 | ST782 | MDR | A clinical *P. aeruginosa* stain | This study |
| ***Escherichia coli*** | | | | |
| DH5α | ST1060 | - | DNA cloning host strain | TransGen |
| BL21 | ST93 | - | Protein expression strain (DE3) | TransGen |
| O157:H7 | - | - | Shiga toxin–producing strain isolated in 1982 | [9] |
| ***Acinetobacter baumanii*** | | | | |
| ATCC 19606 | ST931 | - | A type strain often used in genetic studies, resistant to the sulfonamide compound sulfamethoxazole | [10] |
| AB2 | - | MDR | A carbapenem resistance clinical stain | This study |

**MLST**, multilocus sequencing typing; **MSSA**, methicillin-susceptible *S. aureus*; **MRSA**, methicillin-resistant *S. aureus*; **MSCNS**, methicillin-susceptible coagulase-negative staphylococci; **MRCNS**, methicillin-resistant coagulase-negative staphylococci; -, unknown or not determined.

**Table S2** The MIC values of antimicrobial agents against bacteria.

| **Strains** | **MICs** | | | | | | | |
| --- | --- | --- | --- | --- | --- | --- | --- | --- |
|  | **LysSYL** | |  | **VAN** | |  | **MEM** | |
|  | **μg/mL** | **μM** |  | **μg/mL** | **μM** |  | **μg/mL** | **μM** |
| *S. aureus* XN108 | 128 | 2.34 |  | 12 | 8.08 |  | 64 | 146.28 |
| *S. aureus* USA300 | 32 | 0.58 |  | 1 | 0.67 |  | 8 | 18.29 |
| *S. aureus* N315 | 16 | 0.29 |  | 0.5 | 0.34 |  | 32 | 73.14 |
| *S. aureus* Newman | 32 | 0.58 |  | 1 | 0.67 |  | 2 | 4.57 |
| *S. aureus* 25923 | 32 | 0.58 |  | 2 | 1.35 |  | 2 | 4.57 |
| *E. coli* DH5α | >128 | >2.34 |  | 128 | 86.15 |  | 0.25 | 0.57 |
| *E. coli* O157:H7 | >128 | >2.34 |  | >128 | >86.15 |  | 0.25 | 0.57 |
| *P. aeruginosa* PAO1 | >128 | >2.34 |  | >128 | >86.15 |  | 2 | 4.57 |
| *P. aeruginosa* PA1 | >128 | >2.34 |  | >128 | >86.15 |  | 128 | 292.56 |
| *A. baumannii* 19606 | >128 | >2.34 |  | >128 | >86.15 |  | 2 | 4.57 |
| *A. baumannii* AB2 | >128 | >2.34 |  | >128 | >86.15 |  | >128 | >292.56 |

25923, ATCC 25923; 19606, ATCC 19606; VAN, vancomycin; MEM, meropenem.

**References**

1. Liu L, Peng H, Zhang N, Li M, Chen Z, Shang W, Hu Z, et al. Genomic epidemiology and phenotypic characterization of *Staphylococcus aureus* from a tertiary hospital in Tianjin municipality, Northern China. Microbiol Spectr. 2023;11:e0420922.
2. Cheng H, Yuan W, Zeng F, Hu Q, Shang W, Tang D, et al. Molecular and phenotypic evidence for the spread of three major methicillin-resistant *Staphylococcus aureus* clones associated with two characteristic antimicrobial resistance profiles in China. J Antimicrob Chemother. 2013;68:2453–2457.
3. Kuroda M, Ohta T, Uchiyama I, Baba T, Yuzawa H, Kobayashi I, et al. Whole genome sequencing of meticillin-resistant *Staphylococcus aureus*. Lancet. 2001;357:1225–1240.
4. Zhang X, Hu Q, Yuan W, Shang W, Cheng H, Yuan J, et al. First report of a sequence type 239 vancomycin-intermediate *Staphylococcus aureus* isolate in Mainland China. Diagn Microbiol Infect Dis. 2013;77:64–68.
5. Duthie ES, Lorenz LL. Staphylococcal coagulase: mode of action and antigenicity. J Gen Microbiol. 1952;6:95–107.
6. Diep BA, Gill SR, Chang RF, Phan TH, Chen JH, Davidson MG, et al. Complete genome sequence of USA300, an epidemic clone of community-acquired meticillin-resistant *Staphylococcus aureus*. Lancet. 2006;367:731–739.
7. Treangen TJ, Maybank RA, Enke S, Friss MB, Diviak LF, Karaolis DK, et al. Complete genome sequence of the quality control strain *Staphylococcus aureus* subsp. aureus ATCC 25923. Genome Announc. 2014;2:e01110-14.
8. Shen M, Zhang H, Shen W, Zou Z, Lu S, Li G, et al. *Pseudomonas aeruginosa* MutL promotes large chromosomal deletions through non-homologous end joining to prevent bacteriophage predation. Nucleic Acids Res. 2018;46:4505c14.
9. Li B, Liu H, Wang W. Multiplex real-time PCR assay for detection of *Escherichia coli* O157:H7 and screening for non-O157 Shiga toxin-producing *E. coli*. BMC Microbiol. 2017;17:215.
10. Hamidian M, Hall RM. *Acinetobacter baumannii* ATCC 19606 carries GIsul2 in a genomic island located in the chromosome. Antimicrob Agents Chemother. 2016;61:e01991-16.
